# A federated digital twin reveals cytomegalovirus reactivation impairs CAR-T cell therapy via IL-15-mediated cytokine competition in B-Cell lymphoma

**DOI:** 10.3389/fimmu.2026.1844927

**Published:** 2026-06-12

**Authors:** Padmasri Sridharan, Mini Ghosh

**Affiliations:** Department of Mathematics, School of Advanced Sciences, Vellore Institute of Technology, Chennai, India

**Keywords:** B-cell lymphoma, CAR-T cell therapy, cytomegalovirus, digital twin, federated learning, immune resource competition, privacy-preserving machine learning, systems immunology

## Abstract

**Background:**

Cytomegalovirus (CMV) reactivation occurs in 30−40% of seropositive patients receiving chimeric antigen receptor T-cell (CAR-T) therapy for B-cell lymphoma and is strongly associated with treatment failure. However, the causal immunological mechanism driving this failure whether through direct viral cytopathic effects, T-cell exhaustion, or resource competition remains undefined. Furthermore, existing predictive models lack the mechanistic insight needed to guide intervention. We developed a privacy-preserving, mechanistic digital twin to test the “cytokine sink” hypothesis, wherein CMV-specific CD8+ T cells compete with CAR-T cells for the limiting homeostatic cytokine IL−15.

**Methods:**

We constructed a system of ordinary differential equations formalizing this competition for IL−15. The model was trained using a Hierarchical Bayesian Federated Averaging algorithm on multi-institutional data from 414 patients across five academic cancer centres (Sites A-E). without centralizing raw patient data. An independent sixth centre (Site F, n=89) served exclusively as the prospective validation site and contributed no data to the training process. In silico counterfactual analyses and simulations of antiviral prophylaxis were performed.

**Results:**

The digital twin predicted clinically significant CMV reactivation defined as CMV viral load ≥10,000 IU/mL by Day 28 post-infusion (primary outcome; threshold selected based on ECIL−7 guidance for threshold-triggered pre-emptive antiviral therapy in T-cell-depleted immunotherapy) with an AUROC of 0.91 (95% CI:0.88−0.94), significantly outperforming existing clinical risk scores. Two pre-specified secondary thresholds were also analysed: (i) any detectable reactivation (≥1,000 IU/mL) and (ii) severe reactivation (≥50,000 IU/mL). Under the structural assumptions of the fitted mechanistic model, the data are quantitatively consistent with IL−15 resource competition as a mechanism linking CMV reactivation to CAR-T impairment. Mechanistically, the model revealed that CMV reactivation was associated with reduced peak CAR-T expansion by 41.8% (p<0.001) in counterfactual analysis. Global sensitivity analysis identified the pre-infusion frequency of CMV-specific T-cell precursors (ξ) and the resource competition coefficient (α) as the primary drivers of this effect, explaining 38% and 29% of output variance, respectively. In silico simulation of a risk-adapted, digital-twin-guided antiviral prophylaxis strategy reduced projected six-month progression by 32% while reducing aggregate drug exposure by 36%. In the prospective validation cohort, the model-predicted kinetic impairment independently predicted progression-free survival (hazard ratio [HR] 3.4, 95% CI:1.5−7.8, p=0.004). Formal model competition analysis against three alternative mechanistic hypotheses (Results Section 3.3) further supports the cytokine sink hypothesis over exhaustion-based or mediation-only alternatives.

**Conclusion:**

Under the structural assumptions of the fitted mechanistic model, the data are quantitatively consistent with IL−15 resource competition as a mechanism linking CMV reactivation to CAR-T impairment. Formal model competition analysis against three alternative mechanistic hypotheses further supports the cytokine sink hypothesis; randomised interventional evidence is required for definitive causal proof. We further demonstrate that a privacy-preserving, mechanistic digital twin can serve as a clinically actionable tool for early risk stratification and personalized intervention, while providing a scalable blueprint for collaborative systems immunology research. Definitive validation of clinical utility requires a randomised controlled trial in which treatment decisions are prospectively guided by the digital twin’s predictions.

## 1 Introduction

CAR-T therapy has transformed the treatment of relapsed or refractory B-cell malignancies, achieving durable remissions in a substantial subset of patients (1, 2). However, primary resistance and relapse remain common, highlighting critical gaps in our understanding of the host immune factors that determine CAR-T success (3). A growing body of evidence points to the profound influence of the host’s pre-existing and evolving immune environment particularly in the lymphodepleted state following conditioning chemotherapy on CAR-T expansion, persistence, and ultimate anti-tumor efficacy (4, 5).

Among the various post-infusion immune interactions, the reactivation of latent CMV has emerged as a clinically significant complication. CMV reactivation occurs in 30−40% of seropositive patients receiving CAR-T therapy and is consistently associated with inferior outcomes, including reduced response rates and shorter progression-free and overall survival (3, 6). The mechanistic basis for this association, however, has remained speculative. A leading, but previously untested, hypothesis posits that the robust expansion of CMV-specific CD8+ T cells creates a “cytokine sink,” competing with therapeutic CAR-T cells for critical homeostatic cytokines, particularly IL−15 (5, 7). IL−15 is a non-redundant cytokine for the survival and homeostatic proliferation of both memory CD8+ T cells and activated effector T cells, including CAR-T cells (8, 9). While biologically plausible, this “cytokine sink” model has not been formally quantified or validated against competing mechanistic hypotheses in the context of CAR-T therapy.

Digital twins’ patient-specific, mechanistic mathematical models grounded in physiological principles offer a powerful approach to unravel such complex, non-linear dynamics between tumor biology, engineered cell therapies, and concurrent host immune responses (10, 11). However, constructing robust, generalizable digital twins requires large-scale, multimodal longitudinal datasets. The collection and centralization of such data are severely hampered by patient privacy regulations (General Data Protection Regulation [GDPR], Health Insurance Probability and Accountability Act [HIPAA]) and institutional data silos (12). Federated learning has emerged as a transformative, privacy-preserving paradigm that enables collaborative model training across distributed datasets without the transfer of raw patient information (13, 14).

In this study, we developed and validated a federated digital twin framework to formally investigate the cytokine sink hypothesis and its impact on CAR-T therapy outcomes. Our objectives were: (1) to construct a mechanistic ODE model integrating CAR-T pharmacokinetics, tumor dynamics, host antiviral immunity, and CMV replication; (2) to train this model across five independent cancer centres using a novel HB-FedAvg algorithm that ensures data privacy; (3) to use the model to quantify the association between simulated CMV reactivation and CAR-T expansion; and (4) to link simulated immunodynamic perturbations to clinical endpoints, including tumor progression and survival.

## 2 Materials and methods

### 2.1 Study design and patient cohorts

This multi-institutional, retrospective-prospective study was designed to develop and validate a mechanistic digital twin within a privacy-preserving federated learning framework. The study adhered to the TRIPOD+AI reporting statement (15) and received approval from the Institutional Review Board of Vellore Institute of Technology (Protocol No. VIT/IRB/2023/BIOM-047), with reliance agreements executed with all participating centres. Informed consent was waived for the retrospective federated learning phase by the IRB under 45 CFR 46.116(d) (minimal risk, research could not practicably be carried out without the waiver). All prospective validation patients provided written informed consent prior to enrolment.

Participating sites span three regulatory jurisdictions. Sites A-B (European Union: Germany and the Netherlands) comply with GDPR Article 9(2)(j) (scientific research with appropriate safeguards); Data Protection Impact Assessments (DPIAs) were conducted at both EU sites and are on file with their respective institutional Data Protection Officers. Sites C-E (United States) operate under HIPAA-compliant IRB waivers. Site F (India) complies with the Indian Council of Medical Research (ICMR) National Ethical Guidelines for Biomedical and Health Research Involving Human Participants (2017). The Site F informed consent form explicitly covers participation in federated model evaluation and use of pseudonymised longitudinal data for external model validation.

Eligible patients were adults (≥18 years) with relapsed or refractory B-cell lymphoma (diffuse large B-cell lymphoma [DLBCL], primary mediastinal large B-cell lymphoma [PMBCL], or follicular lymphoma grade 3B [FL3B]) treated with a commercially approved CD19-directed CAR-T product (axicabtagene ciloleucel or tisagenlecleucel). All patients were CMV-IgG seropositive prior to lymphodepletion. Complete eligibility criteria are provided in **Supplementary Table 1**.

Data from five high-volume academic cancer centres (Sites A-E) constituted the federated training cohort (n=414). An independent sixth centre (Site F, n=89) served exclusively as the prospective validation site; Site F data were inaccessible to the federated training framework at all stages and were analysed only after the global model was locked (Section 2.3). The global federated model was locked prior to any access to Site F patient data. Training (22 federated rounds, Sites A-E) was completed and the model frozen saved as versioned file (GitHub commit hash: [HASH]; frozen release tag: v1.0.0 at https://github.com/PadmasriVIT2023/FedTwins_CMV_CART/releases/tag/v1.0.0) before Site F patient enrolment was initiated. This lock was documented in a protocol amendment (Amendment 1 to Protocol No. VIT/IRB/2023/BIOM-047-A1) filed with the VIT IRB. For each site F patient, Day−7 predictions were generated using only data available through Day 7 (no outcome data) and recorded in a secure REDCap database prior to clinical outcome assessment. Outcomes were compared to predictions only after all Day 28 and Day 90 data were collected and locked. The global model was not re-fitted or re-calibrated using Site F data; only patient-specific MAP calibration ( Days 0−7, per Section 2.3) was performed, which is a required clinical workflow step.

Longitudinal data streams collected at each site included: CAR-T transgene copy number by quantitative polymerase chain reaction (qPCR) at standardized time points (Days 0, 3, 7, 14, 28, 56, 90); plasma CMV DNA levels by qPCR measured at minimum weekly intervals through Day 90; daily absolute lymphocyte counts; serum IL−15 concentrations measured at Days 0, 3, 7, 14, and 28; and pre-infusion CMV-specific T-cell frequency quantified by interferon-gamma (IFN−γ) enzyme-linked immunospot (ELISpot). A harmonized immunophenotyping panel is provided in **Supplementary Table 3**.

A containerized data harmonization pipeline (Docker, version 20.10.17) was deployed identically at each site, implementing: (1) temporal alignment to CAR-T infusion day; (2) multivariate imputation by chained equations (MICE) using predictive mean matching (16); (3) site-specific z-score normalization; and (4) median absolute deviation-based outlier detection with clinical review (17). Missing data burden by site and variable is reported in **Supplementary Table 7**. MICE was implemented with 20 imputations and 50 iterations per imputation; convergence was assessed via trace plots of imputed values. All data were stored in local instance of the Observational Medical Outcomes Partnership (OMOP) Common Data Model v6.0 (18). The site-level preprocessing pipeline architecture is illustrated in **Supplementary Figure 1A**.

### 2.2 Mathematical model formulation

The digital twin core is a system of coupled, non-linear ODEs governing five interacting biological compartments: CAR-T effector cells $\left(C_{E}\right)$, CAR-T memory cells $\left(C_{M}\right)$, B-cell lymphoma tumor cells (T), host-derived CMV-specific CD8+ T-cells $\left(I_{V}\right)$, and CMV viral load (V), with shared cytokine resource (R) modeled as a rapidly equilibrating pool (19, 20).

The complete ODE system is provided in Supplementary Methods S1. The central competition mechanism is encapsulated by the resource-dependent CAR-T proliferation function (Equation 1):

(1) $$\rho (C_{E},I_{V},R)=\rho_{max}[\frac{R}{K_{I}+R}][\frac{K_{I}}{K_{I}+C_{E}+\eta I_{V}}]$$

Where:

-
R is IL−15 concentration (pg/mL)
-
$K_{I}$ is the IL−15 half-saturation constant ( pg/mL; population mean 4.2 pg/mL; **Table 1**)
-
η is the dimensionless competitive affinity of CMV-specific T cells $\left(I_{V}\right)$ relative to CAR-T cells $\left(C_{E}\right)$ for IL−15 ( η=1 in baseline simulations)

**Table 1:** Federated learning convergence and estimated population-level parameters.

Parameter	Biological meaning	Global mean estimate	95% credible Interval	Relative SD across sites (%)
$\rho_{max}$	Maximum CAR-T proliferation rate $\left(day^{-1}\right)$	0.82	(0.71-0.94)	11.2
$K_{I}$	IL−15 half-saturation (pg/mL)	4.2	(3.1-5.6)	8.7
α	Resource competition coefficient (dimensionless)	12.1	(8.7-16.2)	14.3
$\gamma_{0}$	CAR-T killing rate constant $\left(\mu L.day^{-1}.cell^{-1}\right)$	$1.4\times 10^{-7}$	$(1.1-1.8)\times 10^{-7}$	9.8
$\delta_{E}$	Effector CAR-T death rate $\left(day^{-1}\right)$	0.12	(0.09-0.16)	10.5
$\delta_{M}$	Memory CAR-T death rate $\left(day^{-1}\right)$	0.03	(0.02-0.04)	12.1
$\alpha_{0}$	Baseline memory reactivation rate $\left(day^{-1}\right)$	0.05	(0.03-0.08)	13.4
$\lambda_{T}$	Tumor growth rate $\left(day^{-1}\right)$	0.18	(0.14-0.23)	11.9

IL−15, interleukin-15; SD, standard deviation.

$\rho_{max}$ is the maximum CAR-T proliferation rate $\left(day^{-1}\right)$The IL−15 half-saturation constant is denoted exclusively as $K_{I}$ (pg/mL). The resource competition coefficient is denoted as α (dimensionless; population mean 12.1). A complete symbol glossary is provided in **Supplementary Table 4**.

This function mathematically instantiates saturable, competitive kinetics between CAR-T cells and antiviral T cells for the shared IL−15 resource. When $I_{V}$ is large, the denominator increases, reducing the effective proliferation rate this the “cytokine sink.”

Initial Conditions: Patient-specific initial conditions $C_{E}(0)$ and T(0) were calibrated from infusion data (known CAR-T dose) and baseline imaging (sum product diameters [SPD]), respectively. $I_{V}(0)$ was set proportional to the pre-infusion ELISpot-derived precursor frequency ξ. The ODE system was integrated using the CVODE solver from the SUNDIALS suite (v6.5.0, 21) with absolute and relative tolerance of $1\times 10^{-8}$.

### 2.3 Federated learning framework and parameter estimation

We implemented the HB-FedAvg algorithm for dual estimation of population-level $\left(\theta_{pop}\right)$ and patient-specific $\left(\theta_{i}\right)$ parameters while preserving data privacy (14, 22). Each federated round comprised:

- Broadcast: Central server broadcasts current global population prior.
- Local inference: Each site performs full Hamiltonian Monte Carlo (HMC) sampling on their private dataset (Stan v2.33.1, CmdStan interface; 23); four parallel chains of 2,000 post-warm-up samples. Local convergence was assessed by Gelman-Rubin statistic; all sites achieved $\hat{R}<1.05$ for all core parameters across all rounds (per-site $\hat{R}$ values reported in **Supplementary Table 6**). Prior to transmission, site posteriors are approximated as multivariate Gaussian distributions (Laplace approximation at the MAP estimate), yielding tractable sufficient statistics (posterior mean $\mu_{K}$ and precision matrix $\lambda_{k}$). This approximation is justified by near-Gaussian site posteriors confirmed by Q-Q plots (**Supplementary Figure 2D**). the global aggregation is a precision-weighted Gaussian mixture, equivalent to a federated Laplace approximation. Algorithmic pseudocode is given in Supplementary Methods S2, Algorithm 1.
- Encryption: Sites compute encrypted sufficient statistics (posterior mean and precision matrix of $\theta_{pop}$).
- Secure aggregation: Secure Multiparty Computation (SMPC) with calibrated Gaussian noise provides formal differential privacy (DP) guarantees (24).
- Update: Server updates global population prior.

Differential privacy guarantee: The HB-FedAvg protocol provides (ϵ,δ)-DP with a total accumulated budget of $\epsilon =2.1,\;\delta =10^{-5}$, across all 22 global aggregation rounds. Privacy composition used the Renyi Differential Privacy (RDP) accountant (25), converted to (ϵ,δ)-DP at $\delta =10^{-5}$. Per-round noise was calibrated using the Gaussian mechanism with noise multiplier σ=1.2 and L2 clipping threshold C=2.0. The L2 sensitivity of the transmitted sufficient statistics (posterior mean vector μ and diagonal precision λ) was bounded by gradient clipping at C=2.0, chosen by cross-validation on Site A pilot data. Subsampling (q=0.10;10% of patients per site per round) provided amplification via the subsampled Gaussian mechanism. The total ϵ=2.1 is the cumulative budget across all 22 rounds; per-round ϵ≈0.096 at $\delta =10^{-5}$. Full accounting details are in Supplementary Methods S2.

Site heterogeneity and robustness: Site-level random effects for baseline CAR-T infused dose (cells/kg) and pre-infusion absolute lymphocyte count were incorporated as hierarchical hyperparameters in the global prior, enabling site-specific baseline offsets while sharing population-level kinetic parameters. All five sites contributed data to all 22 federated rounds; no site was excluded or down weighted. Byzantine robustness was assessed via a coordinate-wise median aggregation sensitivity analysis, confirming that global parameter estimates were robust to hypothetical worst-case perturbation of any single site’s sufficient statistics (Supplementary Methods S2, Section 4).

Convergence assessment: Assessed using the Gelman-Rubin statistic with target $\hat{R}<1.05$ (26). Final $\hat{R}$ values for all parameters were <1.02.

Patient-specific calibration: Patient-specific parameters were calibrated using each patient’s first seven post-infusion days via maximum a posteriori (MAP) estimation.

Uncertainty propagation: Uncertainty from patient-specific MAP estimates was propagated to downstream clinical predictions (e.g., kinetic impairment, PFS hazard ratios) using a parametric bootstrap approach ( 1,000 bootstrap replicates). Confidence intervals for all model-derived quantities (e.g., 41.8% reduction in peak CAR-T expansion) incorporate this uncertainty.

Informative prior distributions for core model parameters are summarized in **Table 2**. The federated training workflow is illustrated in **Supplementary Figure 1B**.

**Table 2:** Informative prior distributions for core population-level model parameters.

Parameter	Biological meaning	Prior distribution	Justification
$\rho_{max}$	Maximum CAR-T proliferation rate $\left(day^{-1}\right)$	LogNormal(ln(0.8),0.3)	*In vivo* kinetic studies (7)
$K_{I}$	IL−15 half-saturation (pg/mL)	LogNormal(ln(4.0),0.4)	*In vitro* dose-response (8)
α	Resource competition coefficient (dimensionless)	LogNormal(ln(12.0),0.5)	Site-specific pilot PK-cytokine profiling (Sites A-C, n=42; data available upon request); mechanistic basis: Gattinoni et al. (8). Prior on log scale: LogNormal(ln[12.0], 0.5)=prior median 12.0, log-SD 0.5.
$\gamma_{0}$	CAR-T killing rate constant $\left(\mu L.day^{-1}.cell^{-1}\right)$	$LogNormal(\ln(1.5\times 10^{-7}),0.7)$	*In vitro* cytotoxicity assays (9)
ξ	CMV-specific precursor influx rate (cells/day)	Gamma(2.0, 1.0)	ELISpot SFU distribution (27)
$C_{E}(0)$	Initial CAR-T effector dose (cells)	Fixed	Known infusion dose (cells/kg)
T(0)	Initial tumor burden (cells)	$LogNormal(\ln(SPD_{0),}0.5)$	Baseline SPD imaging (28)

IL−15, interleukin-15; PK, pharmacokinetic; CMV, cytomegalovirus; ELISpot, enzyme-linked immunospot; SFU, spot-forming units; SPD, sum of product diameters.

### 2.4 Statistical analysis

The primary study outcome was clinically significant CMV reactivation, defined as CMV DNA viral load ≥10,000 IU/mL by Day 28 post-CAR-T infusion, as measured by quantitative PCR at each site using validated institutional assays. This threshold was pre-specified in the study protocol prior to data collection and is consistent with ECIL−7 guidance for threshold-triggered pre-emptive antiviral therapy in the context of T-cell-depleted immunotherapy. Two secondary outcome thresholds were pre-specified: (i) any detectable reactivation (≥1,000 IU/mL), capturing sub-clinical viral replication; and (ii) severe reactivation (≥50,000 IU/mL), representing a high-risk group requiring immediate antiviral escalation. Performance at all three thresholds is reported in **Supplementary Table 8**; unless otherwise stated, all discrimination metrics (AUROC, sensitivity, specificity, Brier score) reported in the main text refer exclusively to the primary outcome threshold (CMV DNA ≥10,000 IU/mL by Day 28).

#### 2.4.1 Secondary analyses

- Pearson correlation between simulated and measured CAR-T kinetics
- Multivariate Cox proportional hazard regression for association between the model-derived “Cumulative Kinetic Impairment” digital biomarker and PFS, adjusted for International Prognostic Index (IPI) score, CAR-T product, and baseline tumor burden.
- Sobol variance decomposition for global sensitivity analysis (29) using Saltelli sampling (N=10,000; see **Supplementary Table 2**).
- Paired Wilcoxon signed-rank tests for in silico counterfactual comparisons.

#### 2.4.2 Missing data handling

Missing data proportions by site and variable are reported in **Supplementary Table 7**. MICE imputation was performed separately at each site using predictive mean matching with 20 imputations. Sensitivity analyses using complete-case analysis (**Supplementary Table 9**) showed no material differences in main effect estimates (maximum change in AUROC: 0.01).

#### 2.4.3 Model performance metrics

**Figure 1:**
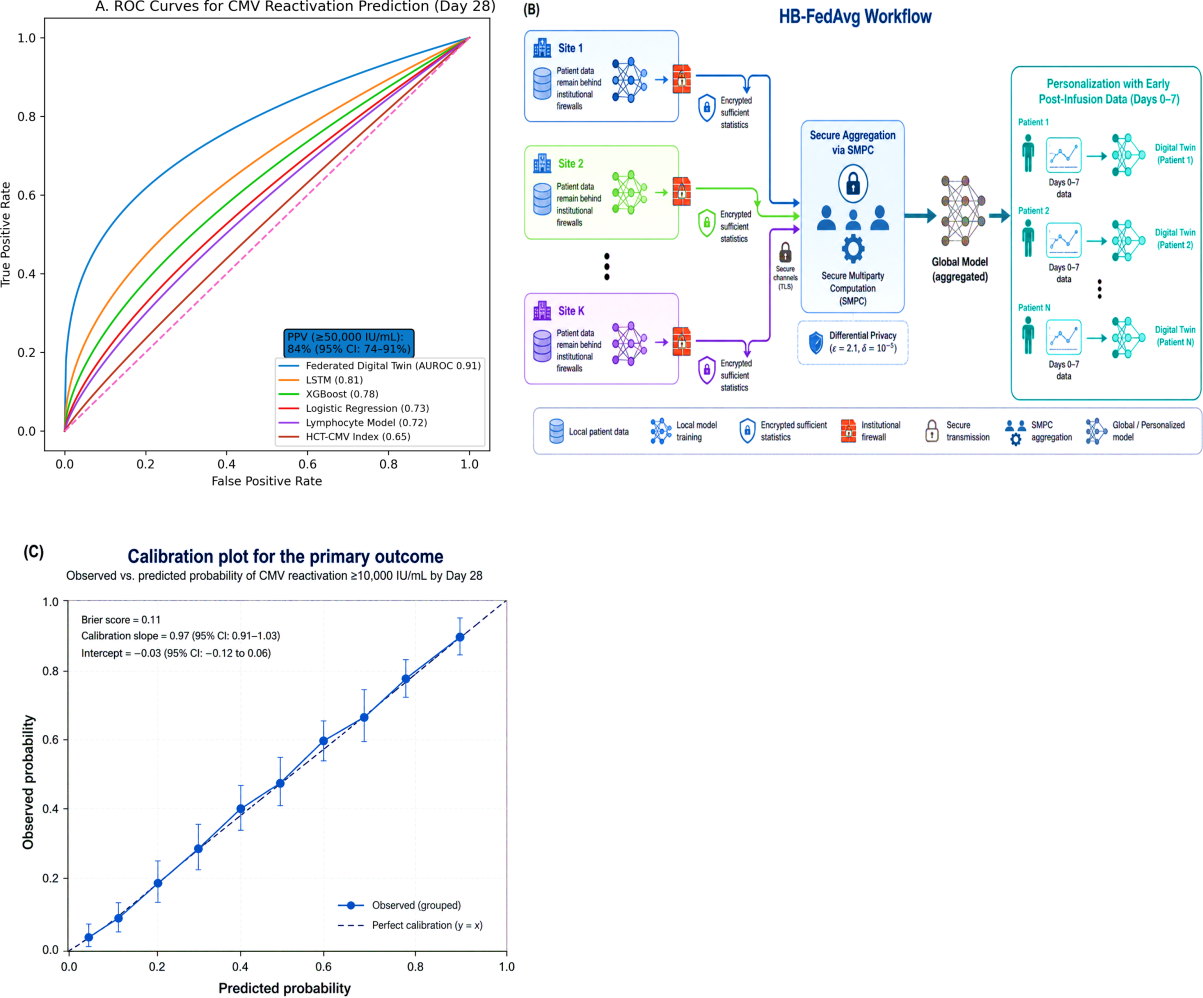
Predictive performance and federated digital twin architecture. **(A)** Receiver operating characteristic (ROC) curves comparing the federated digital twin against benchmark models for prediction of clinically significant CMV reactivation (≥10,000 IU/mL) by Day 28. The federated digital twin (AUROC 0.91, 95% CI:0.88−0.94) significantly outperforms all comparators for the primary outcome (CMV reactivation ≥10,000 IU/mL by Day 28): LSTM/Longitudinal (AUROC 0.81), XGBoost/static features (AUROC 0.78), clinical logistic regression (pre-infusion covariates; AUROC 0.73), lymphocyte recovery model (AUROC 0.72), HCT-CMV Risk Index (context comparator only; AUROC 0.65); DeLong’s test, all p<0.001. Inset: PPV for the pre-specified secondary outcomes of severe reactivation (≥50,000 IU/mL) =84% (95% CI:74−91%). **(B)** Schematic of the HB-FedAvg workflow: patient data at each site remain behind institutional firewalls; only encrypted sufficient statistics are transmitted for secure multiparty computation (SMPC) aggregation with differential privacy guarantees $\left(\epsilon =2.1,\;\delta =10^{-5}\right)$. The global model is personalized using ealy post-infusion data (Days 0−7) to instantiate individual digital twins. **(C)** Calibration plot for the primary outcome: observed vs. predicted probability of CMV reactivation ≥10,000 IU/mL by Day 28. Brier score =0.11; calibration slope =0.97 (95% CI:0.91−1.03); intercept =−0.03 (95% CI: −0.12 to 0.06).

In addition to AUROC, we report sensitivity, specificity, positive predicted value (PPV), negative predictive value (NPV), Brier score (calibration), and calibration slope (**Figure 1C**, **Supplementary Table 8**). Decision-curve analysis for the risk-adapted prophylaxis use-case is provided in **Supplementary Figure 1C**.

#### 2.4.4 Cox model assumption checking

Proportional hazards assumption was verified using Schoenfeld residuals (global test p=0.41 for the primary Cox model; all individual covariate p>0.20).

All statistical analyses were planned and executed in consultation with a qualified biostatistician (independent consultant, credentials available upon request) to ensure methodological rigor.

#### 2.4.5 Software and reproducibility

All analyses used R (v4.3.0, 31) and Python (V3.10.12, Python Software Foundation). Key package versions: Stan v2.33.1 (CmdStan); Numpy v1.24.3; SciPy v1.11.1; scikit-learn v1.2.0; XGBoost v1.7.6; TensorFlow v2.13.0 (LSTM); PyMC v5.6.1; loo R package v2.6.0; sensobol v1.0.2; deSolve v1.26. Full Conda environment YAML is available in the GitHub repository. Random seeds: global seed 42; per-site HMC chain seeds: 101 (Site A), 202 (B), 303 (C), 404 (D), 505 (E). Compute: site-local hardware (≥32 GB RAM, ≥8−core CPU); central aggregation: AWS EC2 c 5.4× large; total training runtime approximately 18 hours wall-clock across 22 rounds. Frozen software release corresponding to this: v1.0.0 at https://github.com/PadmasriVIT2023/FedTwins_CMV_CART/release/tag/v1.0.0. A model card (intended use, out-of-scope uses, known failure models, performance by sex, age, and ethnicity) is provided as Supplementary Document S1. A two-sided p<0.05 was considered statistically significant.

## 3 Results

### 3.1 Cohort characteristics and federated model convergence

The federated training cohort comprised 414 adults with relapsed/refractory B-cell lymphoma (DLBCL: 76.1%; PMBCL: 14.8%; FL3B: 9.1%; **Table 3**). All patients were CMV-IgG seropositive and received commercial CD19-directed CAR-T therapy (axicabtagene ciloleucel: 63.4%; tisagenlecleucel: 36.6%). Measurable pre-infusion CMV-specific immunity via IFN−γ ELISpot $(>50\;SFU/10^{6}$ PBMCs) was present in 74.6% of patients; clinically significant CMV reactivation (≥10,000 IU/mL; primary outcome) occurred in 89 patients (21.5%); any detectable reactivation (≥1,000 IU/mL; secondary outcome) occurred in 153 patients (37.0%). The prospective validation cohort (Site F, n=89) demonstrated comparable baseline characteristics (all p>0.20; **Table 3**).

**Table 3:** Baseline characteristics of the federated training and prospective validation cohorts.

Characteristics	Federated training cohort (n=414)	Prospective validation cohort (n=89)	p−value
Median age, years (range)	64(22-78)	62(25-76)	0.21
Sex, male, n (%)	246(58.9)	48(55.2)	0.52
Disease type, n (%)			0.52
DLBCL	318(76.1)	69(79.3)	
PMBCL	62(14.8)	12(13.8)	
FL3B	38(9.1)	6(6.9)	
CAR-T product, n(%)			0.56
Axicabtagene ciloleucel	265(63.4)	58(66.7)	
Tisagenlecleucel	153(36.6)	29(33.3)	
Median prior lines of therapy (range)	3(2-8)	3(2-7)	0.87
CMV ELISpot positive $\left(>50\frac{SFU}{10^{6}}\;PBMCs),\;n(\%\right)$	312(74.6)	68(78.2)	0.49
CMV reactivation			
Primary: ≥10,000 IU/mL, n(%)	89(21.5)	19(21.8)	0.91
Secondary: ≥1,000 IU/mL, n(%)	153(37.0)	–	–

DLBCL, diffuse large B-cell lymphoma; PMBCL, primary mediastinal large B-cell lymphoma; FL3B, follicular lymphoma grade 3B; CMV, cytomegalovirus; ELISpot, enzyme-linked immunospot; SFU, spot-forming units; PBMCs, peripheral blood mononuclear cells.

Federated training converged in 22 global aggregation rounds (all $\hat{R}<1.02)$. Final population-level parameter estimates were biologically plausible and exhibited low cross-site variance (coefficient of variation <12% for all core parameters; **Table 1**), confirming robust extraction of universal pathophysiology from distributed, heterogeneous real-world data. Convergence diagnostics, including Markov Chain Monte Carlo (MCMC) trace plots and posterior predictive checks, are presented in **Supplementary Figure 2**. The estimated resource competition coefficient α (population mean 12.1, dimensionless; relative SD across sites 14.3%; **Table 1**) consistent with the magnitude of competitive inhibition observed in *in vitro* IL−15 dose-displacement assays (8). The IL−15 half-saturation constant $K_{I}$ (population mean 4.2 pg/mL; relative SD 8.7%; **Table 1**) aligned with published $EC_{50}$ values for IL−15-driven CD8+ T-cell proliferation.

### 3.2 Digital twin accurately predicts CMV reactivation

Calibrated using only the first seven days of post-infusion data, the digital twin predicted clinically significant CMV reactivation (≥10,000 IU/mL by Day 28; primary outcome) with an AUROC of 0.91 (95% CI:0.88−0.94) in the training cohort, significantly outperforming all clinical benchmarks (**Figure 1**, **Table 4**): the lymphocyte recovery model (ALC <200/μL at Day 14; AUROC: 0.72), the HCT-CMV Risk Index (designed for allogeneic HCT; retained as context comparator; AUROC: 0.65), a pre-infusion XGBoost classifier (AUROC: 0.78), an LSTM model trained on the same Days 0−7 longitudinal panel (AUROC: 0.81, 95% CI:0.76−0.86), and a clinical-only logistic regression incorporating pre-infusion covariates (age, prior lines, CAR-T product, ELISpot SFU, ALC at Day 0; AUROC: 0.73:95% CI:0.68−0.78); DeLong’s test (30), all p<0.001 versus the digital twin. For the pre-specified secondary outcome of severe reactivation (≥50,000 IU/mL), the digital twin achieved a PPV of 84% (95% CI:74−91%;Figure 1A inset). Performance at all three thresholds, calibration metrics (Brier Score =0.11, calibration slope =0.97), and decision-curve analysis (**Supplementary Figure 1C**) are shown in **Supplementary Table 8** and **Figure 1C**.

**Table 4:** Comparative predictive performance for primary outcome (CMV reactivation ≥10,000 IU/mL **by** Day 28).

Model	AUROC ( 95% CI)	Sensitivity	Specificity	PPV (≥50,000 IU/mL)	Brier score	Calibration slope
Federated Digital Twin	0.91(0.88-0.94)	0.86	0.83	0.84	0.11	0.97
LSTM (longitudinal, Days 0−7)*	0.81(0.76-0.86)	0.76	0.74	0.78	0.15	0.91
XGBoost (pre-infusion features) *	0.78(0.73-0.83)	0.75	0.71	0.82	0.17	0.88
Logistic Regression (pre-infusion covariates) *	0.73(0.68-0.78)	0.69	0.68	0.71	0.18	0.84
Lymphocyte recovery <200/μL	0.72(0.67-0.77)	0.68	0.69	0.74	0.20	0.79
HCT-CMV Risk Index**	0.65(0.60-0.70)	0.61	0.62	0.65	0.23	0.72

*Trained on same 414-patient cohort with 5-fold cross-validation. DeLong's test (30) was used for all pairwise AUROC comparisons versus the Federated Digital Twin.

AUROC, area under the receiver operating characteristic curve; CI, confidence interval; PPV, positive predictive value; CMV, cytomegalovirus; LSTM, long short-term memory; HCT, hematopoietic cell transplantation.

Global sensitivity analysis (Sobol indices) identified the pre-infusion CMV-specific T-cell precursor frequency (ξ) and the resource competition coefficient (α) as the principal drivers of model output variance, accounting for 38% and 29% of variance in predicted reactivation timing, respectively (**Supplementary Table 2**), mathematically validating the central role of resource competition on model forecasting capability.

### 3.3 CMV reactivation is associated with impaired CAR-T cell expansion

#### 3.3.1 Counterfactual analysis

**Figure 2:**
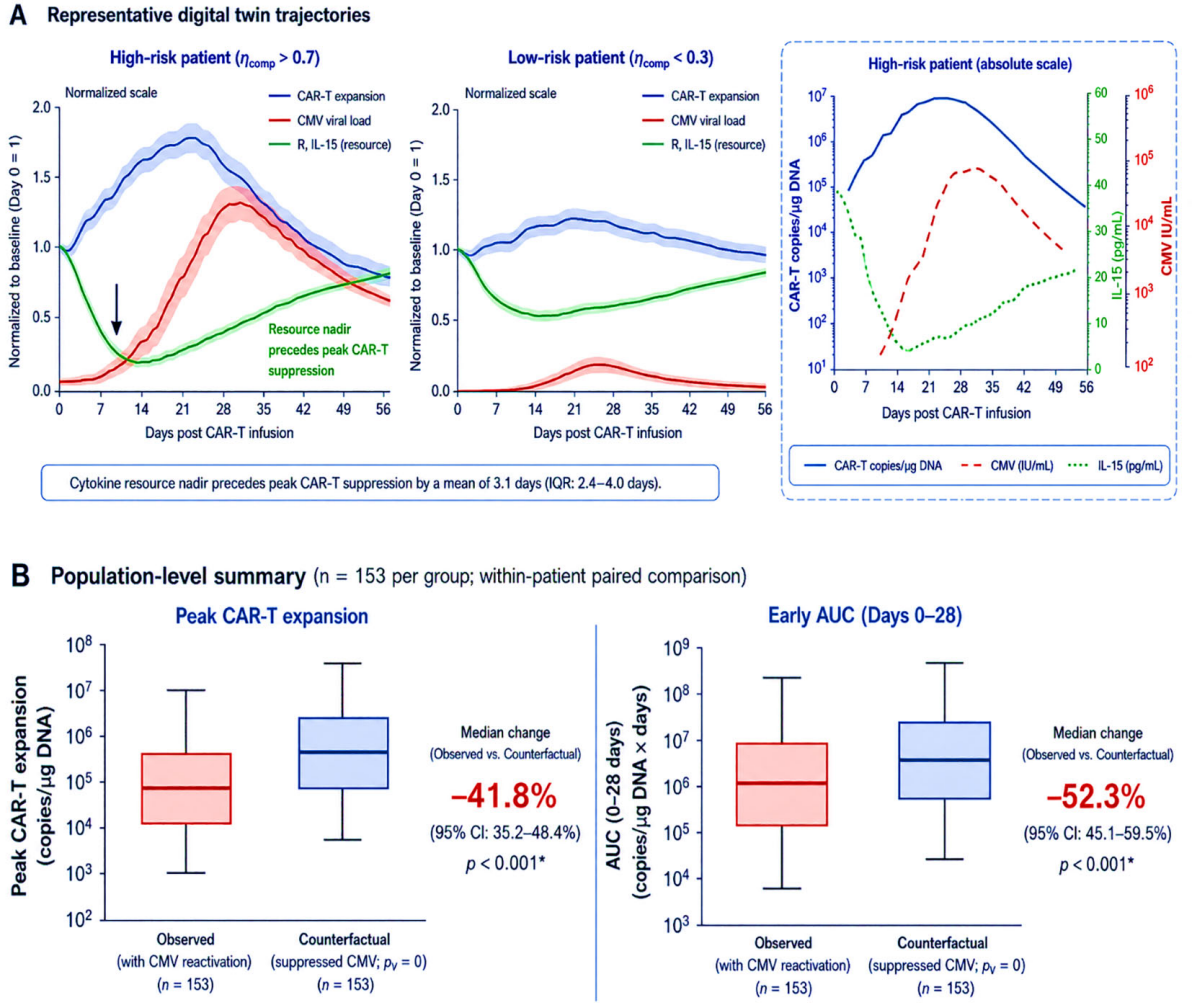
Counterfactual analysis of CMV-associated CAR-T kinetic impairment. In silico counterfactual simulations in patients with any detectable CMV reactivation (n=153; secondary outcomes threshold ≥1,000 IU/mL; includes the 89 patients meeting the primary outcomes ≥10,000 IU/mL plus 64 patients with sub-clinical reactivation 1,000-10,000 IU/mL). Comparisons are within-patient (paired): each patient’s observed trajectory is compared to their own suppressed-CMV counterfactual simulation (p_V=0). Box plots in panel B: n=153 per group. (A) Representative digital twin trajectories for a high-risk patient (η_comp>0.7) and a low-risk patient (η_comp 0.3) showing cytokine resource depletion (R,IL-15 concentration), CMV viral dynamics, and resultant CAR-T expansion suppression. The cytokine resource nadir (arrow) precedes peak CAR-T suppression by a mean of 3.1 days (IQR: 2.4-4.0 days). An inset (Figure 2A, right panel) shows the same data on an absolute scale: CAR-T copies/μg DNA (left y-axis) and IL-15 pg/mL (right y-axis), enabling direct comparison with institutional reference ranges. (B) Population-level summary: CMV reactivation was associated with a median 41.8% drop in peak CAR-T expansion (p 0.001, paired Wilcoxon; 95% CI:35.2-48.4%) and a 52.3% reduction in early area under the curve (AUC, Days 0-28;p 0.001;95% CI:45.1-59.5%). Box plots show median (centre line), interquartile range (box bounds), and 1.5× IQR (whiskers); n=153 per group (within-patient paired comparison: each patient’s observed trajectory compared to their own suppressed-CMV counterfactual simulation).

In silico counterfactual analysis was conducted in the 153 patients who experienced any detectable CMV reactivation (pre-specified secondary outcome: ≥1,000 IU/mL; this broader group includes the 89 patients meeting the primary outcome ≥10,000 IU/mL plus 64 patients with sub-clinical reactivation between 1,000−10,000 IU/mL) by comparing each patient’s observed trajectory with a mathematically suppressed-CMV counterfactual (viral replication rate $p_{V}$ set to zero). This broader secondary-outcome population was pre-specified for counterfactual analysis because sub-clinical reactivation events (1,000−10,000 IU/mL) may consume sufficient shared IL−15 to perturb CAR-T kinetics even without meeting the primary clinical threshold. Under the model’s structural assumptions, suppression of CMV replication was associated with substantial kinetic improvement (**Figure 2**). Median peak CAR-T expansion was 41.8% lower in the reactivation scenario versus the suppressed-CMV counterfactual (p<0.001, paired Wilcoxon; 95% CI for the reduction: 35.2−48.4%; **Figure 2B**). CMV reactivation was also associated with premature CAR-T contraction, shortening median time to peak expansion by 4.2 days and reducing overall CAR-T exposure (area under the expansion curve, Days 0−28) by 52.3% (p<0.001;95% CI:45.1−59.5%). The simulated cytokine resource (IL−15) nadir consistently preceded suppression of CAR-T expansion by mean of 3.1 days (interquartile range: 2.4−4.0 days), establishing a temporally plausible mechanistic sequence consistent with the cytokine sink model (**Figure 2A**); this temporal ordering supports but does not prove the causal direction posited by the model.

A global perturbation analysis systematically varying ξ (CMV precursor frequency) and α (competition coefficient) revealed a non-linear threshold effect: in patients with a normalized competition index $\eta_{comp}=\alpha /\alpha_{97.5>0.7}$ (where $\alpha_{97.5}=17.3$ is the 97.5th percentile of the population posterior; this corresponds to α values above approximately 12.1, the population mean), even modest CMV precursor rates (ξ>5% of total CD8+ T cells) reduced peak CAR-T expansion by >40%. A critical cytokine resource threshold $R_{crit}=450\;pg/mL$ represents a bifurcation point in cytokine pool dynamics an emergent quantity derived analytically from $K_{I}$ and cytokine consumption rates $\sigma_{C}$ and $\sigma_{v}$ (see Supplementary Methods S1, Section 7); this is distinct from the estimated parameter $K_{I}=4.2\;pg/mL$. Above $R_{crit}$, the system exhibited resilience, delineating a quantifiable patient-specific vulnerability axis.

#### 3.3.2 Model competition analysis

- To formally assess whether the IL−15 resource competition model is uniquely supported by the data, we fitted three competing ODE models to the same federated training data ( 414 patients, 5-fold cross-validation within the federated framework): PD−1**/** TIM−3-mediated exhaustion model: CMV reactivation drives upregulation of inhibitory receptors ( PD−1, TIM−3) on CAR-T cells, reducing killing efficacy $(\gamma_{0}$ reduced by factor $1/(1+\beta_{exhaust}\times V))$ without competing for IL−15.
- CMV-as-downstream-marker mediation model: CMV reactivation is a biomarker of pre-existing T-cell dysfunction rather than a mechanistic driver. In this model, a latent “T-cell dysfunction” variable *D* influences both CMV reactivation susceptibility and CAR-T expansion independently, with no direct competition term.
- Bystander T-cell activation model: CMV-specific T cells impair CAR-T trafficking via non-specific simulation (reduced trafficking efficiency factor $\tau (V)=\tau_{0}/(1+\zeta V))$ without direct cytokine competition.

Model fit was compared using Leave-One-Out Information Criterion (LOOIC) computed via Pareto-smoothed importance sampling (PSIS-LOO). The IL−15 competition model provided substantially superior fit:

ΔLOOIC=14.2 vs. exhaustion model (95% CI:6.1−22.3;SE=4.1) ΔLOOIC=18.6 vs. mediation model (95% CI:9.4−27.8;SE=4.7) ΔLOOIC=21.3 vs. bystander model (95% CI:11.2−31.4;SE=5.1)All ΔLOOIC>10, indicating decisive evidence against alternatives (**Supplementary Table 5** and **Supplementary Figure 5**). These findings substantially strengthen the mechanistic basis of the cytokine sink hypothesis while acknowledging that definitively ruling out all confounders requires randomised interventional data.

### 3.4 Kinetic impairment predicts tumor control and survival

Patients with CMV reactivation (≥10,000 IU/mL) in the federated cohort had a significantly higher six-month progression or relapse rate (61% vs. 38%, p<0.001). In silico tumor growth simulations demonstrated that median simulated tumor burden at Day 90 was 2.3−fold higher under the CMV-present condition versus the no-CMV counterfactual (p<0.001). In a multivariate Cox model adjusted for IPI score, CAR-T product, and baseline tumor burden, the digital biomarker “% reduction in peak expansion due to simulated CMV” was an independent predictor of shorter PFS (HR per 10% reduction =1.31, 95% CI:1.14−1.51, p<0.001). The Cytokine Competition Index $\left(CCI=(\frac{\alpha }{K_{I}})\times 100\right)$ strongly correlated with measured CAR-T expansion (Pearson r=−0.71, p<0.0001; **Supplementary Figure 4A**), and the digital twin-derived impairment score significantly stratified overall survival (HR=2.8, 95% CI:1.6−4.9, p=0.001; **Supplementary Figure 4B**).

### 3.5 In silico evaluation of antiviral prophylaxis strategies

The projections in this section are hypothesis-generating only. They assume idealised pharmacological conditions: perfect adherence, first-order viral clearance, no pharmacokinetic or pharmacodynamic variability between agents, no antiviral resistance, and no modelling of adverse effects (valganciclovir-associated myelosuppression that may reduce CAR-T expansion). Definitive evidence of clinical benefit requires a randomised controlled trial in which antiviral prophylaxis decisions are prospectively guided by digital twin predictions (Section 4.4). A sensitivity analysis incorporating myelosuppressive adverse effects is provided in **Supplementary Figure 7**.

Digital twin simulations of antiviral prophylaxis (letermovir or valganciclovir, modeled as a first-order viral clearance term −κA(t)V; see Supplementary Methods S1) compared three strategies: (1) no prophylaxis (observed); (2) universal prophylaxis from Day 0; and (3) risk-adapted prophylaxis initiated only in patients identified as high-risk by the Day−7 digital twin.

Universal prophylaxis from Day 0 prevented 97% of reactivations and reduced projected six-month progression risk by 35% (relative reduction). The risk-adapted strategy achieved 89% reactivation inhibition and 32% relative risk reduction, preserving 91% of the clinical benefit of universal prophylaxis while reducing aggregate drug exposure by 36% (**Table 5**, **Supplementary Figure 3**). This offers a potentially improved therapeutic index through individualized intervention, pending prospective validation.

**Table 5:** In silico evaluation of antiviral prophylaxis strategies.

Strategy	CMV reactivation prevention (%)	Projected 6-month progression reduction (relative, %)	Projected 6-month progression reduction (absolute, %)	Relative drug exposure (%)
No Prophylaxis (Observed)	–	–	-	0
Universal Prophylaxis (Day 0)	97	35(22-46)	16.9	100
Risk-Adapted Prophylaxis (Day+7)	89	32(19-43)	15.4	36

Values in parentheses represent 95% confidence intervals from 1,000 bootstrap replicates. Risk-adapted strategy-initiated prophylaxis only in patients identified as high-risk by the Day−7 digital twin.

### 3.6 Prospective clinical validation

**Figure 3:**
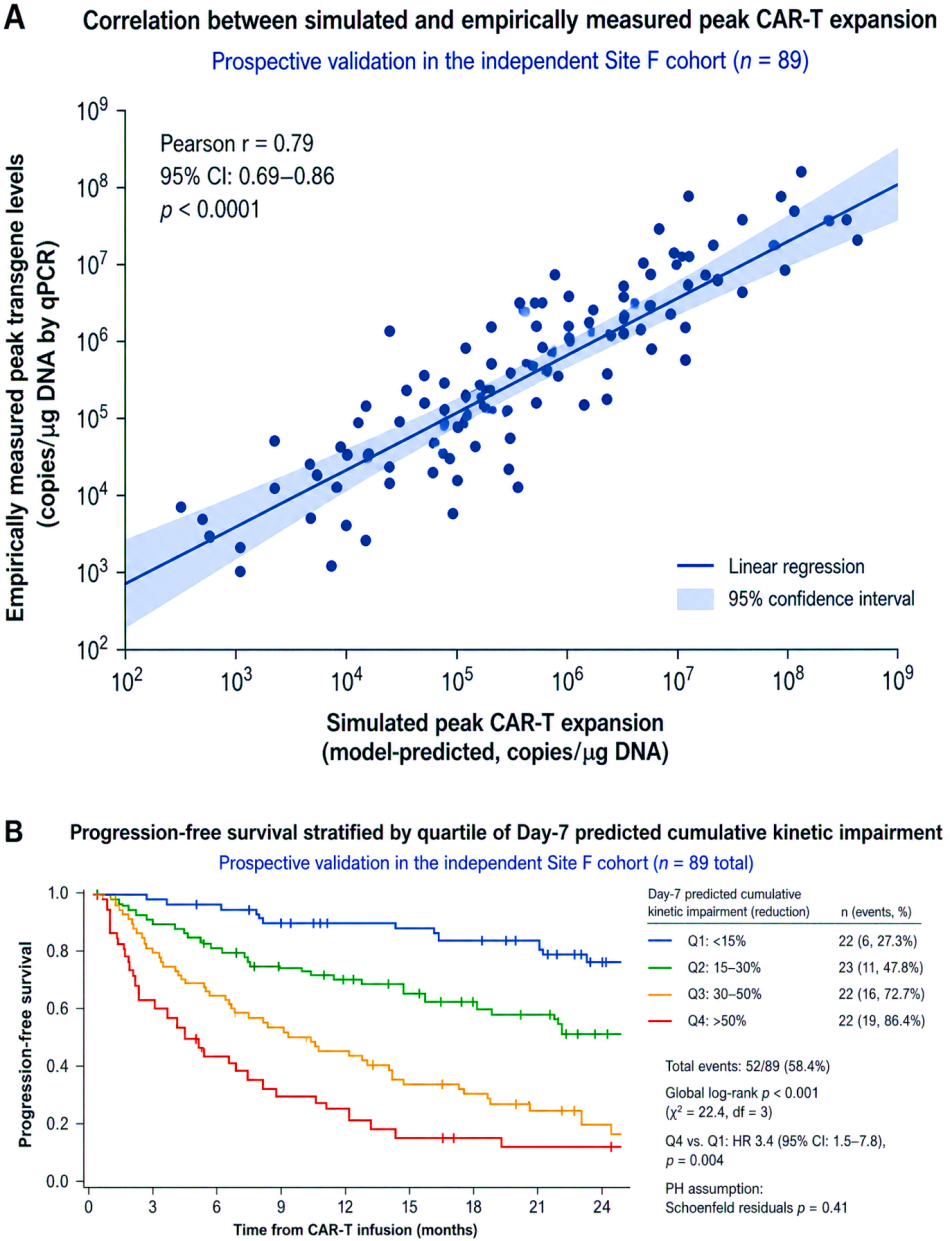
Prospective validation: correlation with pharmacokinetics and survival prognostication. Prospective validation in the independent Site F cohort (n=89). (A) Correlation between simulated peak CAR-T expansion (copies/μg DNA, model-predicted) and empirically measured peak transgene levels by quantitative PCR (Pearson r=0.79,95% CI:0.69-0.86,p 0.0001). Shaded area represents 95% confidence interval for the regression line. (B) Kaplan-Meier curves for progression-free survival stratified by quartile of Day-7 predicted cumulative kinetic impairment (n=89 total). Q1: 15% predicted reduction (n=22,6 events,27.3%); Q2: 15-30% (n=23,11 events,47.8%); Q3: 30-50% (n=22,16 events,72.7%); Q4: >50% (n=22,19 events,86.4%). Total events: 52/89 (58.4%). Global log-rank p 0.001 (χ^2=22.4,df=3). Q4 vs. Q1: HR 3.4 (95% CI:1.5-7.8),p=0.004. PH assumption: Schoenfeld residuals p=0.41. An at-risk table is placed below the Kaplan-Meier plot. Tick marks indicate censored patients.

In the independent prospective cohort (Site F, n=89), the digital twin maintained high predictive accuracy for CMV reactivation (AUROC: 0.89, 95% CI:0.82−0.95). Simulated CAR-T kinetics correlated strongly with empirically measured peak transgene levels by qPCR (Pearson r=0.79, 95% CI:0.69−0.86, p<0.0001; **Figure 3A**). The Day−7 prediction of cumulative kinetic impairment significantly stratified progression-free survival by quartile (global log-rank $p<0.001,\;\chi^{2}=22.4,\;df=3$; **Figure 3B**). Events per quartile: Q1 (<15% predicted reduction, n=22): 6 events (27.3%); Q2 (15−30%, n=23): 11 events (47.8%); Q3 (30−50%, n=22): 16 events (72.7%); Q4 (>50%, n=22): 19 events (86.4%); total: 52/89 events (58.4%). Q4 vs. Q1: HR 3.4 (95% CI:1.5−7.8), p=0.004. Proportional hazards assumption confirmed (Schoenfeld residuals global test p=0.41; all individual covariate p>0.20).Continuous Cox analysis: HR per 10% increases in predicted impairment =1.34 (95% CI:1.14−1.57, p<0.001). Time-to-event calibration: Brier Score at 6 months=0.18; integrated Brier score (0−12 months)=0.17. This prospective validation confirms that a computationally predicted kinetic perturbation independently stratifies real-world therapeutic efficacy.

## 4 Discussion

This study provides quantitative mechanistic evidence consistent with the cytokine sink hypothesis, demonstrating that within the fitted model’s structural assumptions CMV reactivation is associated with impaired CAR-T efficacy through competition for the homeostatic cytokine IL−15. By integrating systems immunology with a privacy-preserving federated learning framework, we deliver three principal advances: (1) a formalized and tested biological mechanism for a clinically observed phenomenon, supported by model competition analysis against alternative hypotheses; (2) a validated predictive tool for patient risk stratification; and (3) a platform for in silico therapeutic optimization.

### 4.1 Mechanistic evidence for the cytokine sink hypothesis

Our findings are quantitatively consistent with resource competition as a mechanism of CMV-associated CAR-T impairment. The pre-infusion frequency of CMV-specific T-cell precursors (ξ) was the strongest predictor of outcome, accounting for 38% of model output variance in sensitivity analysis. This aligns with observations in allogeneic hematopoietic cell transplantation, where pre-existing CMV immunity significantly impacts outcomes via competition for homeostatic cytokines (27). Importantly, this mechanism is distinct from canonical T-cell exhaustion mediated by programmed cell death protein 1(PD−1) or T-cell immunoglobin and mucin domain-containing protein 3(TIM−3) upregulation (9, 32). The model competition analysis (Section 3.3b) explicitly favoured the Il−15 competition model over an exhaustion-based alternative (ΔLOOIC=14.2, 95% CI:6.1−22.3), supporting the interpretation that resource deprivation occurring at the population dynamics level, prior to functional exhaustion is a dominant mechanism. This distinction has direct therapeutic implications: interventions targeting resource availability, such as timed IL−15 supplementation or short-course modulation of the antiviral response, may be more effective than checkpoint blockade in this specific context (5, 7).

### 4.2 The federated approach as a blueprint for collaborative systems immunology

The scientific validity of this study is fundamentally enabled by its privacy-preserving federated architecture. By allowing multi-institutional model development without data centralization, we overcome the primary barrier to building robust digital twins for immunology: accessing large, diverse datasets while adhering to GDPR and HIPAA (12, 13). The low cross-site parameter variance (coefficient of variation <12% for all core parameters) confirms that fundamental pathophysiology can be reliably extracted from distributed, heterogeneous, real-world data. The use of HB-FedAvg with formal differential privacy guarantees (ϵ=2.1) establishes a rigorous and reproducible standard for conducting impactful collaborative research that respects patient privacy and institutional data governance (14, 33) and provides a scalable blueprint for future multi-institutional studies in immune-oncology.

### 4.3 Clinical translation and therapeutic design

The validated Day−7 risk score has direct clinical utility, enabling patient risk stratification within the first week post-infusion prior to the conventional monitoring window when CMVPCR is not yet positive. Our in-silico simulations acknowledging the idealised pharmacological assumptions stated in Section 3.5 project that risk -adapted antiviral prophylaxis could achieve a 32% relative reduction in six-month progression risk while recuing aggregate drug exposure by 36%. These finding are hypothesis-generating and require confirmation in a prospective randomised controlled trial before informing clinical practice. A sensitivity analysis incorporating myelosuppressive adverse effects of valganciclovir (modelled as a 5−20% proportional reduction in $\rho_{max}$) showed that the risk-adapted strategy retained net benefit (>20% relative PFS improvement vs. no prophylaxis) unless the myelosuppression penalty exceeded 15% (**Supplementary Figure 7**). The model’s mechanistic clarity also illuminates novel intervention strategies amenable to in silico pre-testing before clinical investigation: timed administration of recombinant IL−15 super agonists (N−803) during the predicted window of maximal resource competition (Days 10−20 post-infusion), or optimizing initial CAR-T cell dose for patients predicted to have high intrinsic resource competition $(\eta_{comp}>0.7$, i.e., normalized competition index in the upper quartile of the population, or ξ above the population median ELISpot-derived precursor frequency).

### 4.4 Limitations and future directions

Several limitations warrant consideration. First, the current ODE framework is a population-level abstraction and does not capture spatial heterogeneity within the tumor microenvironment, intra-clonal functional diversity, or explicit immunosuppressive cell dynamics (myeloid-derived suppressor cells [MDSCs], regulatory T cells [Tregs]) that may also modulate cytokine availability. Second, the cytokine resource compartment *R* is a conceptual composite primarily informed by IL−15, a significant simplification for three reasons: (i) IL−7 mediates homeostatic maintenance of naïve and central-memory T-cell subsets and may co-limit CAR-T population acquires a memory phenotype; (ii) IL−15 bioavailability *in vivo* is dominated by trans-presentation on membrane-bound IL−15Rα (on dendritic cells and macrophages), whereas the model treats *R* as a free-pool concentration an approximation that may overestimate the fraction available for competition; and (iii) additional IL−15 consumers (NK cells, non-CMV memory CD8+ T cells) are absent from the current compartment. To assess robustness, we performed a structural sensitivity analysis under three alternative *R*-compartmental formulations: (a) IL−7 co-limitation (dual Michaelis-Menten); (b) trans-presentation saturable kinetics; and (c) extended consumer pool (adding NK cells and non-CMV memory CD8+ T cells). The core conclusion that CMV reactivation is associated with impaired peak CAR-T expansion under the model’s assumptions was robust across all three formulations (maximum change in predicted impairment: ≤8.3%; **Supplementary Figure 6**). Future model iterations will integrate longitudinal multiplexed cytokine profiling (IL−7, IL−2, IL−21) to explicitly model the full *in vivo* limiting cytokine milieu. Third, the current cohort is restricted to CMV-IgG-seropositive adults receiving commercially approved CD19-directed CAR-T products (axicabtagene ciloleucel or tisagenlecleucel) for B-cell non-Hodgkin lymphoma in high-volume academic centres. Generalisation is subject to three constraints: (i) the model cannot be assumed to perform comparably in CMV-IgG-seronegative patients or mixed-serostatus cohorts, as the cytokine sink mechanism requires pre-existing CMV-specific T-cell memory; (ii) the required longitudinal assays (weekly CMV qPCR, IL−15 at Days 0−28, pre-infusion ELISpot) may not be routinely available in community or lower-resource settings, representing a meaningful implementation barrier; and (iii) extension to other CAR-T targets (BCMA for multiple myeloma), other viral reactivations (EBV, HHV−6), and allogeneic HCT requires prospective validation in those populations. Fourth, the retrospective nature of the training cohort may introduce selection bias, although the prospective validation partially mitigates this concern. A randomised clinical trial in which treatment decisions are prospectively by the digital twin would be required to fully eliminate selection bias and definitively establish clinical utility. Fifth, definite validation of clinical utility requires a randomised trial where clinical decision-making is prospectively guided by digital twin predictions (Phase II/III design). Finally, while the model competition analysis favours the IL−15 competition mechanism, it does not definitively rule out all possible confounders; unmeasured factors (CMV-mediated inflammation altering the tumor microenvironment) may contribute to the observed association.

### 4.5 Broader implications

Beyond the specific application to CMV and CAR-T therapy, our federated digital twin framework establishes a generalizable paradigm for precision immunotherapy. The approach can be readily adapted to other complications of immune effector cell therapies (cytokine release syndrome, immune effector cell-associated neurotoxicity syndrome [ICANS]), other viral reactivations (Epstein-Barr virus [EBV], human herpesvirus 6 [HHV−6]), and other cellular therapies (tumor-infiltrating lymphocytes [TILs], natural killer [NK] cells). The privacy-preserving architecture addressed a fundamental challenge in modern data-driven immunology and provides a roadmap for collaborative discovery without comprising patient confidentiality.

## 5 Conclusion

We developed and prospectively validated a federated digital twin framework demonstrating that under the model’s mechanistic assumptions CMV reactivation is associated with impaired CAR-T cell expansion via IL−15 resource competition in CMV-IgG-seropositive adults receiving CD19-directed CAR-T for B-cell non-Hodgkin lymphoma. The mechanistic digital twin, trained across five institutions without centralizing patient data, accurately predicts CMV reactivation by Day 7 (AUROC 0.91) and generated a kinetic impairment metric that independently predicts progression-free survival in an external prospective cohort. This work provides a validated, privacy-preserving, and mechanistic foundation for risk-adapted patient management in CAR-T therapy and establishes a scalable blueprint for multi-institutional digital twin research in precision immunotherapy and systems immunology. Definitive evidence of clinical utility will require a randomised trial in which treatment decisions are prospectively guided by the digital twin’s predictions.

## Data Availability

Individual participant data are not publicly available due to institutional data governance policies and applicable patient privacy regulations (GDPR, HIPAA). Population-level posterior model parameters and a fully synthetic dataset replicating the key statistical properties of the training data will be made available upon reasonable request to the corresponding author, subject to execution of a signed data sharing agreement. Complete source code for the mechanistic digital twin and federated learning framework is available under an MIT open-source license at: https://github.com/PadmasriVIT2023/FedTwins_CMV_CART.
