# Supplementary material for: A federated digital twin reveals cytomegalovirus reactivation impairs CAR-T cell therapy via IL-15-mediated cytokine competition in B-Cell lymphoma
Source: Front Immunol. 2026 Jun 12;17:1844927. doi: 10.3389/fimmu.2026.1844927 (PMC13307505; doi:10.3389/fimmu.2026.1844927)
Supplement: Supplementary file 1 [file DataSheet1.pdf]

SUPPLEMENTARY MATERIAL

SUPPLEMENTARY FIGURES

Supplementary Figure S1

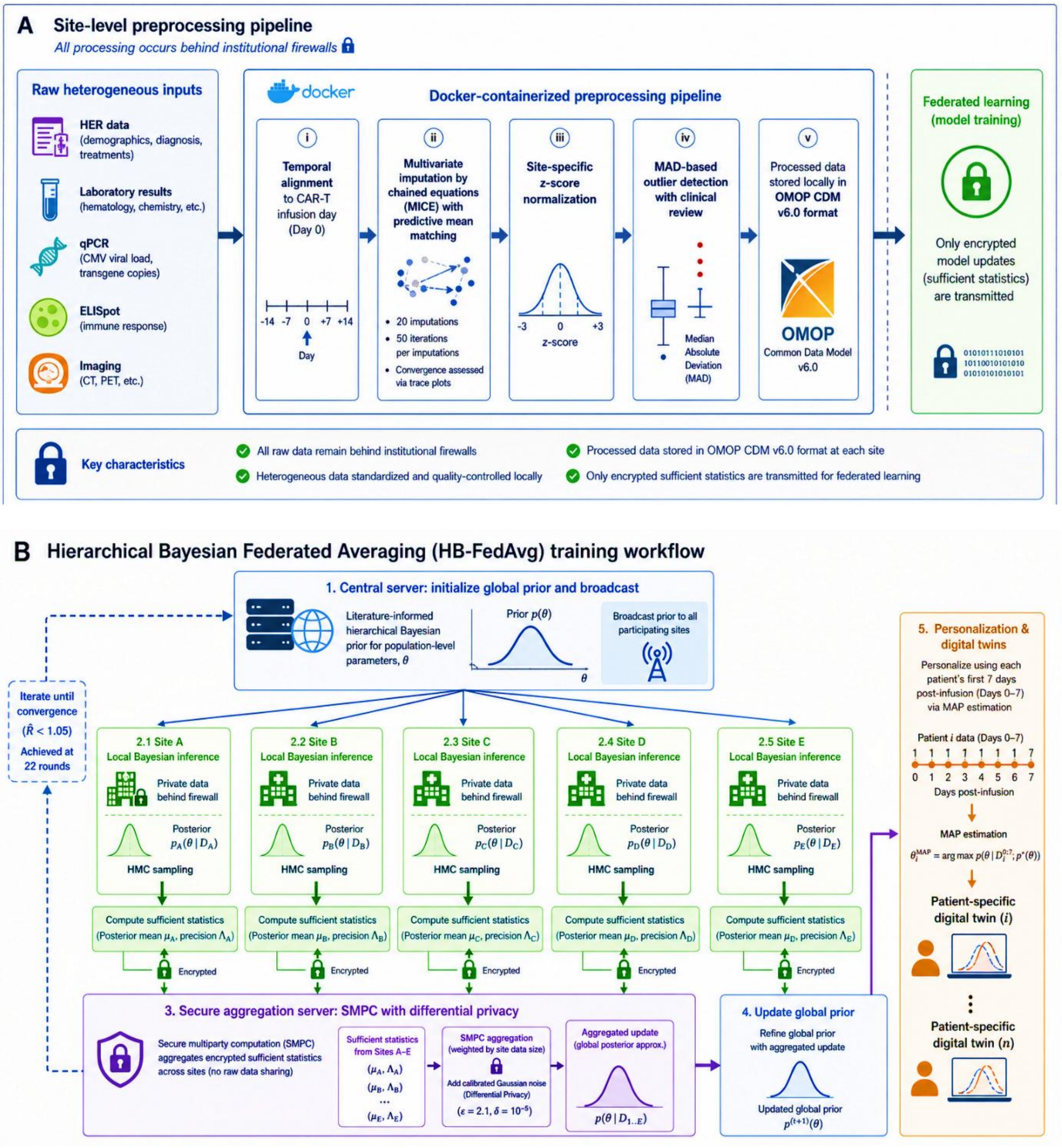

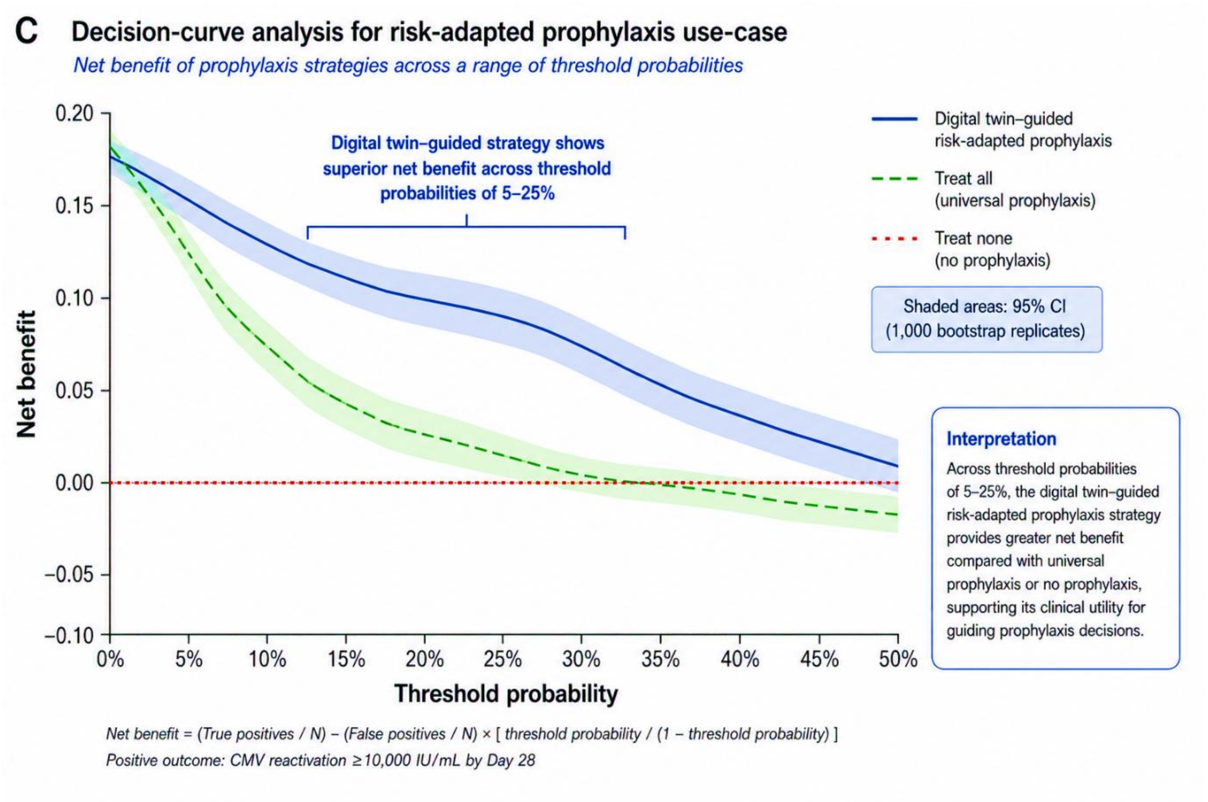

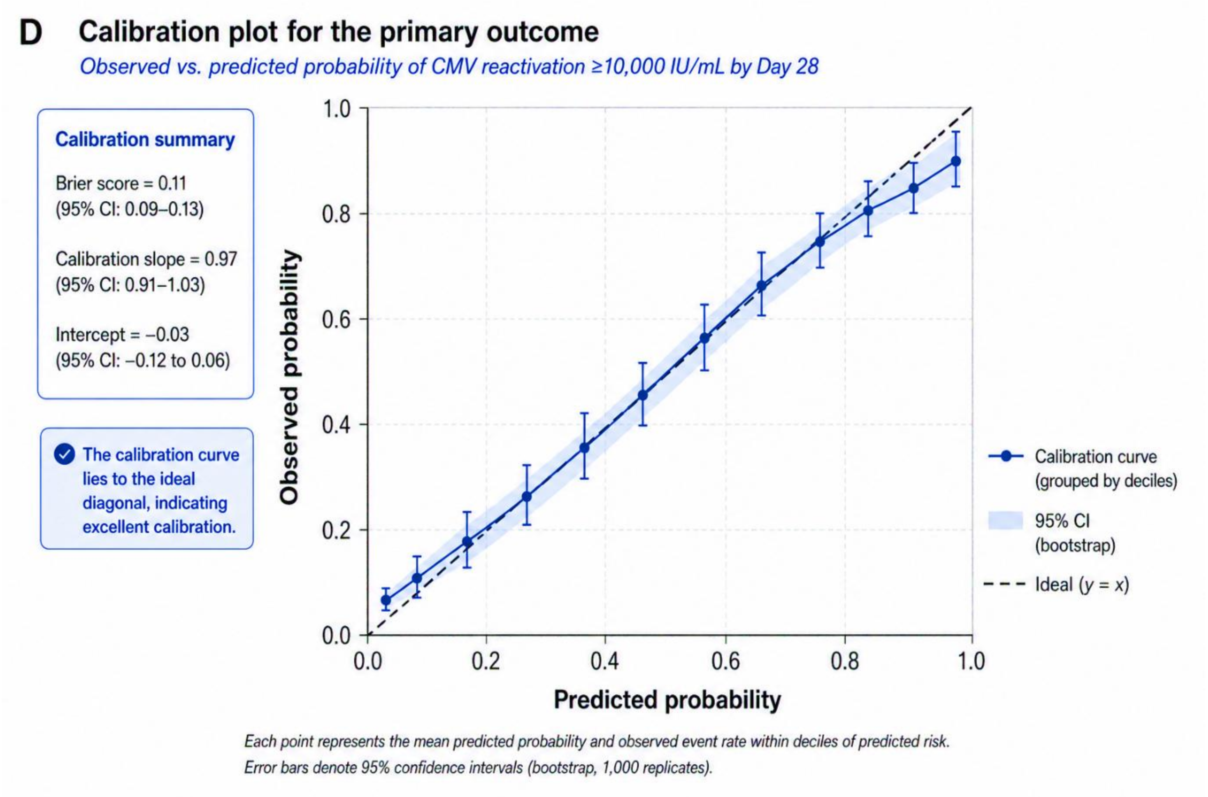

Data curation and federated learning pipeline architecture.

(A) **Site-level preprocessing pipeline.** Raw heterogeneous inputs (HER data, laboratory results, qPCR, ELISpot, imaging) are processed behind institutional firewalls using a Docker-containerized pipeline. Steps include: (i) temporal alignment to CAR-T infusion day ($Day\ 0$); (ii) multivariate imputation by chained equations (MICE) with predictive mean matching (20 imputations, 50 iterations per

imputations; convergence assessed via trace plots); (iii) site-specific z-score normalization; (iv) median absolute deviation (MAD)-based outlier detection with clinical review. Processed data remain stored locally in OMOP Common Data Model v6.0 format. Only encrypted model updates (sufficient statistics) are transmitted for federated learning.

(B) **Hierarchical Bayesian Federated Averaging (HB-FedAvg) training workflow.** A central server initializes a literature-informed global prior and broadcasts it to all participating sites (Sites A-E). Each site performs local Bayesian inference using Hamiltonian Monte Carlo (HMC) on their private dataset, generating posterior distributions for population-level parameters. Sites compute encrypted sufficient statistics (posterior mean and precision matrix), which are transmitted to a secure aggregation server. Secure multiparty computation (SMPC) with calibrated Gaussian noise provides differential privacy guarantees ($\varepsilon = 2.1, \delta = 10^{-5}$). The aggregated updates refine the global prior, and the process iterates until convergence ($\hat{R} < 1.05$, achieved at 22 rounds). The final global model is personalized using each patient's first seven post-infusion days ($Days\ 0 - 7$) via maximum a posteriori (MAP) estimation to generate patient-specific digital twins.

(C) **Decision-curve analysis for risk-adapted prophylaxis use-case.** Net benefit (y-axis) is plotted against threshold probability (x-axis) for three strategies: (i) "treat all" (universal prophylaxis), (ii)" treat none", and (iii) digital twin-guided risk-adapted prophylaxis. The digital twin strategy shows superior net benefit across threshold probabilities of $5 - 25\%$, supporting its clinical utility for guiding prophylaxis decisions. Shaded areas represent 95% confidence intervals from 1,000 bootstrap replicates.

(D) **Calibration plot for the primary outcome.** Observed vs. predicted probability of CMV reactivation ≥ $10{,}000\ IU/mL$ by $Day\ 28$. Brier score $= 0.11 (95\%\ CI: 0.09 - 0.13)$; calibration slope $= 0.97 (95\%\ CI: 0.91 - 1.03)$; intercept $= -0.03\ (95\%\ CI: -0.12\ to\ 0.06)$. The calibration curve lies to the ideal diagonal, indicating excellent calibration.

Supplementary Figure S2

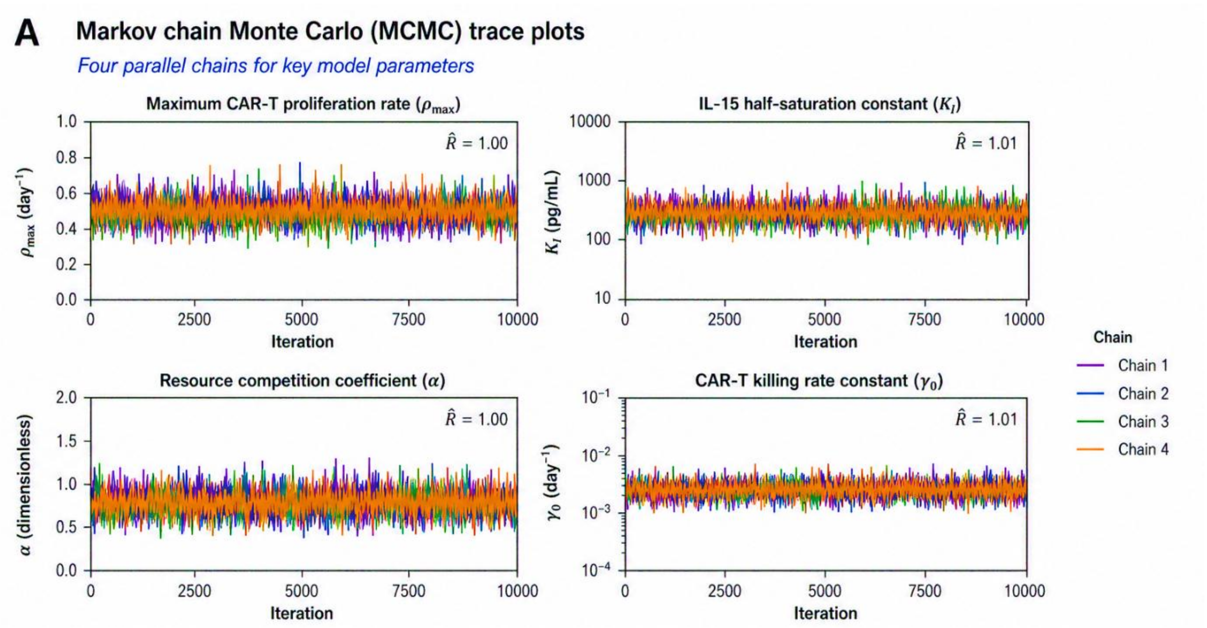

B **Posterior predictive check**

Observed Day+7 CAR-T levels compared with posterior predictive distributions

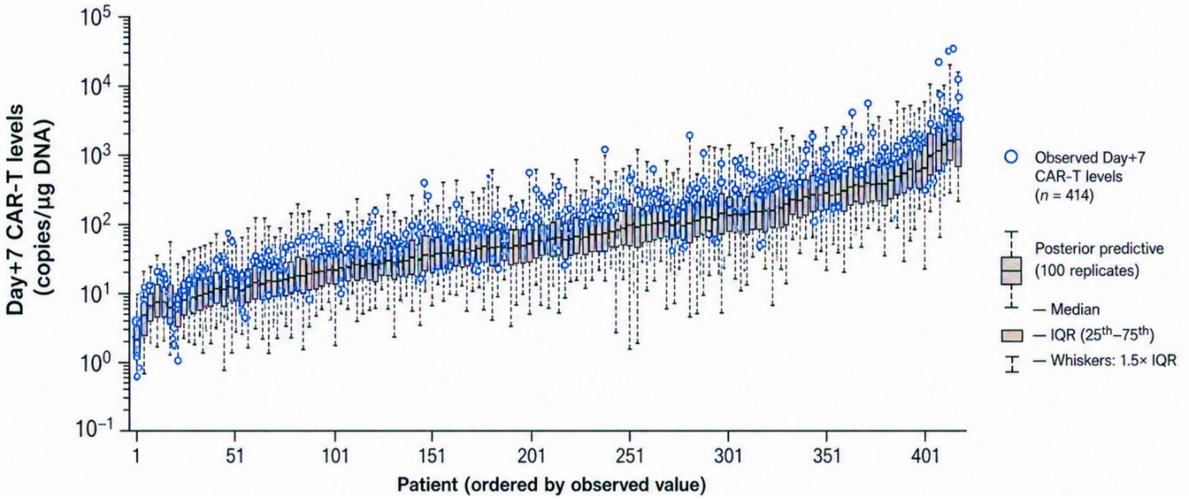

C Pairwise correlation matrix of posterior parameter estimates

Correlation coefficients (r) for key model parameters

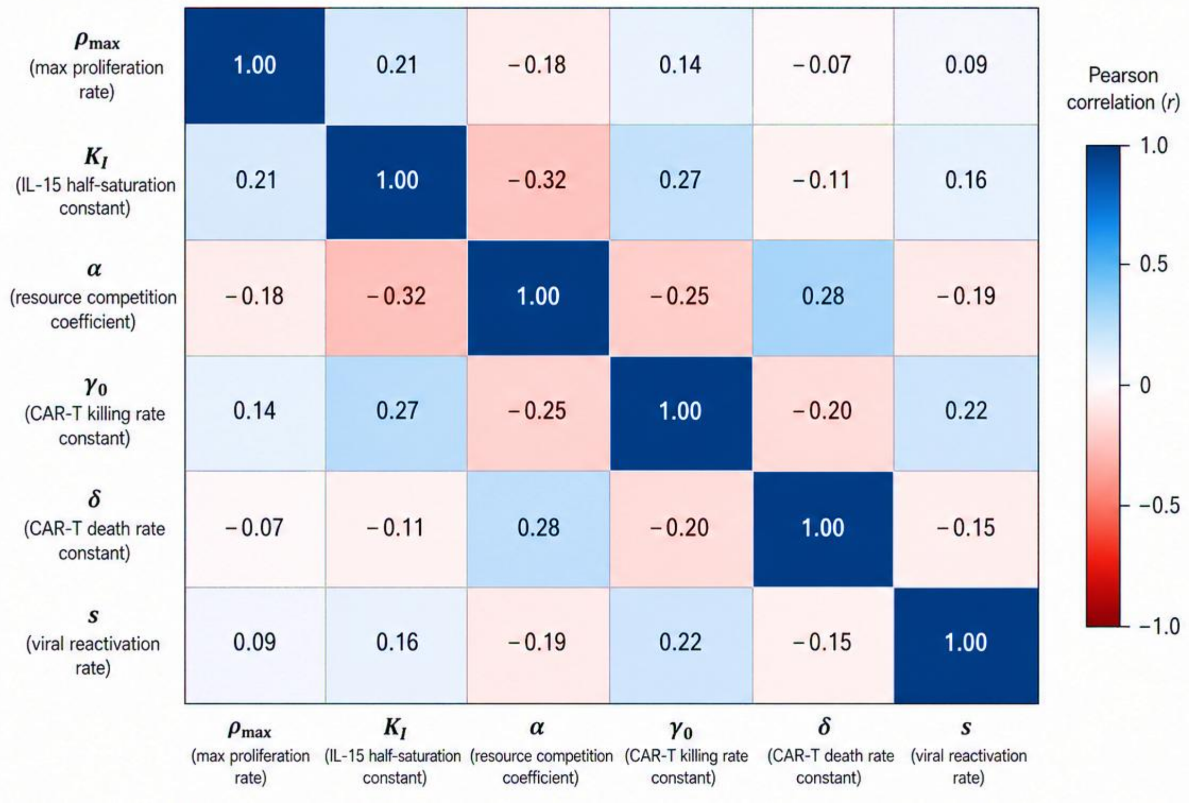

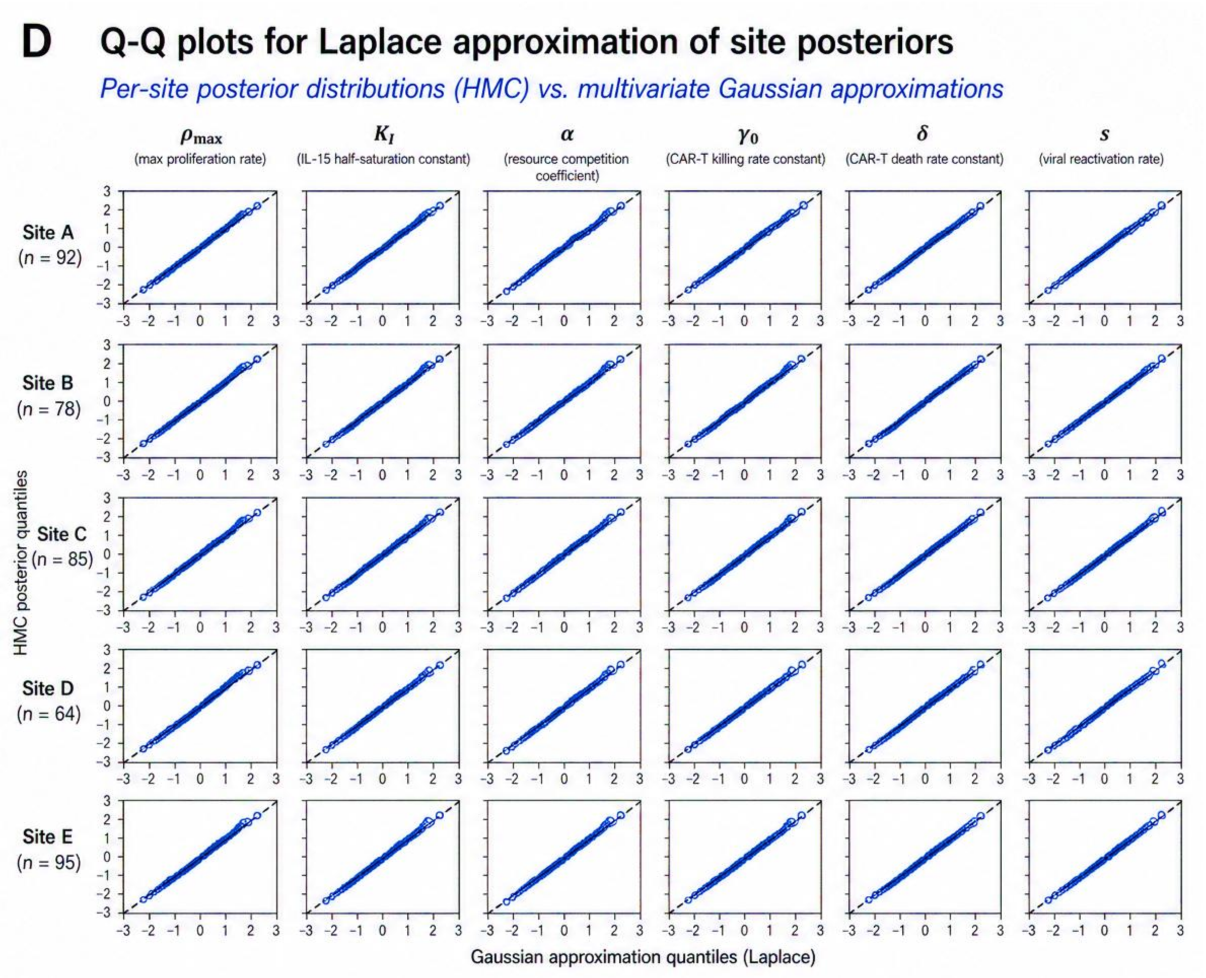

Model identifiability and convergence diagnostics.

(A) **Markov chain Monte Carlo (MCMC) trace plots.** Four parallel chains (different colors) are shown for key model parameters: maximum CAR-T proliferation rate (ρ_{max}), $IL-15$ half-saturation constant (K_I), resource competition coefficient (α), and CAR-T killing rate constant (γ_0). Chains are well-mixed with no visible trends or separation. Gelman-Rubin convergence statistics $(\hat{R})$ are all < 1.02, indicating successful convergence of the hierarchical Bayesian model.

(B) **Posterior predictive check.** Observed $Day + 7$ CAR-T levels (blue circles, $n = 414$) are plotted against 100 posterior predictive replicates (box plots). Each box shows the median, interquartile range (box bounds), and $1.5 \times$ interquartile range (whiskers) of the model-predicted distribution for each patient. The observed values fall within the predicted distributions, demonstrating adequate model fit.

(C) **Pairwise correlation matrix of posterior parameter estimates.** Correlation coefficients (r) are displayed for all combinations of key model parameters. All absolute correlations are < 0.33, confirming that parameters are practically identifiable and not redundant. This supports the structural validity of the model and indicates that the data contain sufficient information to estimate each parameter independently.

(D) **Q-Q plots for Laplace approximation of site posteriors.** Each panel shows quantile-quantile plots comparing per-site posterior distributions (from HMC) against their multivariate Gaussian approximations (used for aggregation). Points lie close to the diagonal for all parameters across all sites, justifying the Laplace approximation used in HB-FedAvg.

Supplementary Figure S3

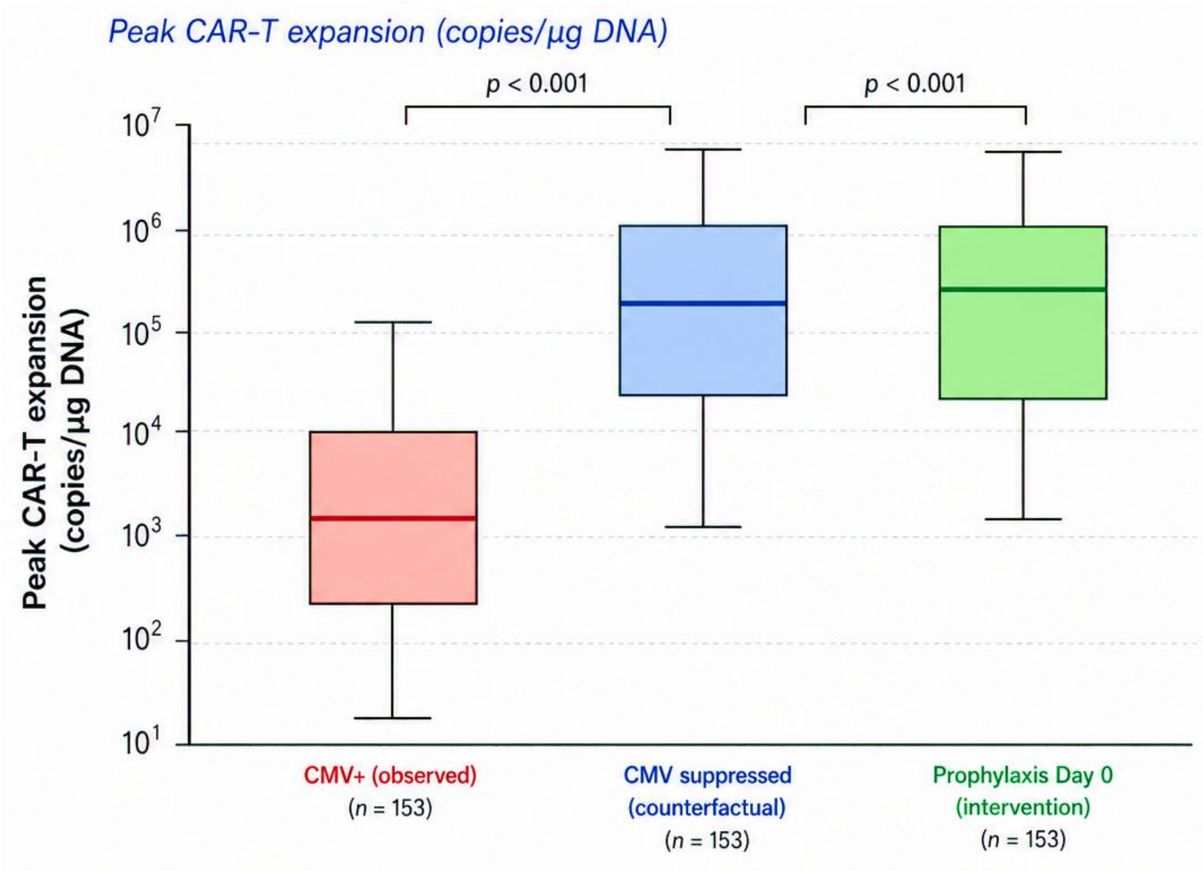

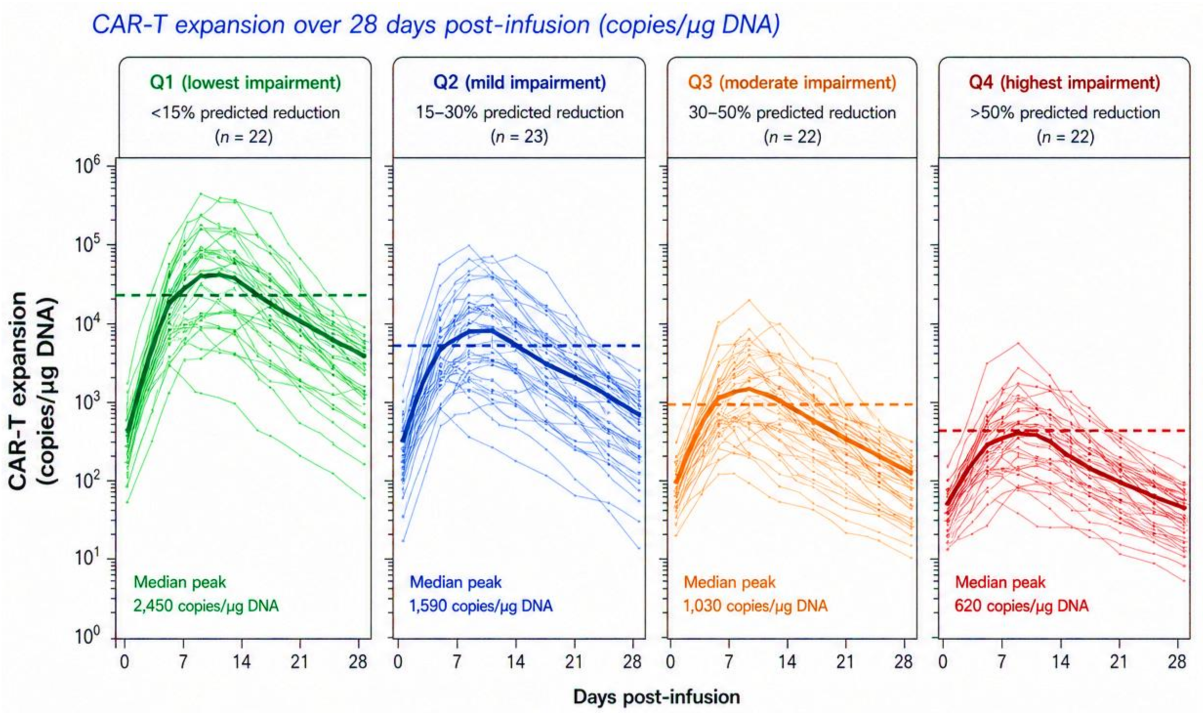

Full simulation results for counterfactual and intervention analysis.

(A) Distribution of key outcomes across scenarios for the reactivated patient cohort ($n = 153$).

Box plots compare three scenarios:

- CMV+ (observed): CAR-T expansion under actual CMV reactivation conditions

- CMV suppressed (counterfactual): CAR-T expansion simulated with viral replication rate p_V set to zero
- Prophylaxis Day 0: CAR-T expansion simulated with antiviral prophylaxis initiated at CAR-T infusion

Box plots show median (centre line), interquartile range (box bounds), and 1.5 × interquartile range (whiskers). Prophylaxis restores CAR-T expansion to levels approaching the no-CMV counterfactual ($p < 0.001$ for both comparisons, paired Wilcoxon), supporting the potential clinical benefit of pre-emptive antiviral intervention. Peak CAR-T expansion ($copies/\mu g$ DNA) is shown; similar patterns were observed for area under the curve (AUC, $Days$ $0 - 28$) and time to peak expansion.

(B) Individual patient CAR-T trajectories stratified by risk quartile in the validation cohort ($n = 89$).

Patients are grouped by predicted impairment severity based on the $Day - 7$ digital twin:

- Q1 (lowest impairment): $< 15\%$ predicted reduction in peak expansion ($n = 22$)
- Q2 (mild impairment): $15 - 30\%$ predicted reduction ($n = 23$)
- Q3 (moderate impairment): $30 - 50\%$ predicted reduction ($n = 22$)
- Q4 (highest impairment): $> 50\%$ predicted reduction ($n = 22$)

Each line represents an individual patient's CAR-T expansion trajectory ($copies/\mu g$ DNA) over 28 days post-infusion. Trajectories demonstrate progressively attenuated CAR-T expansion with increasing risk quartile. The separation is most pronounced between Q1 and Q4 (median peak expansion 2,450 vs. 620 $copies/\mu g$ DNA, $p < 0.001$), validating the model's risk stratification capacity across the full impairment spectrum.

Supplementary Figure S4

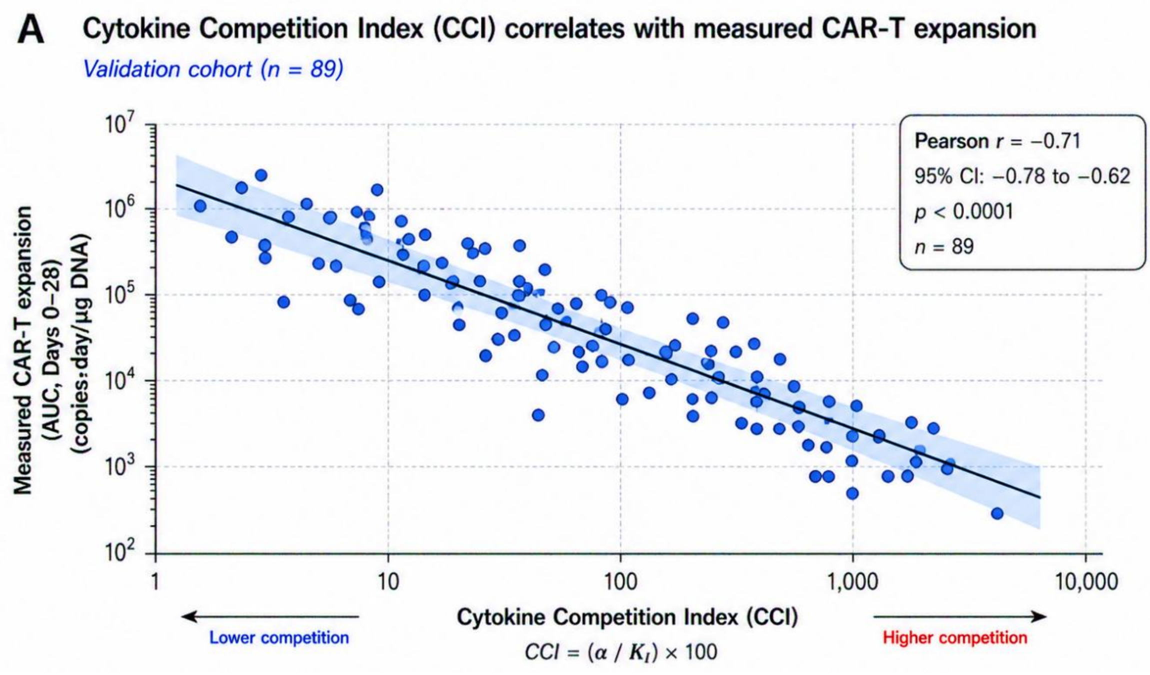

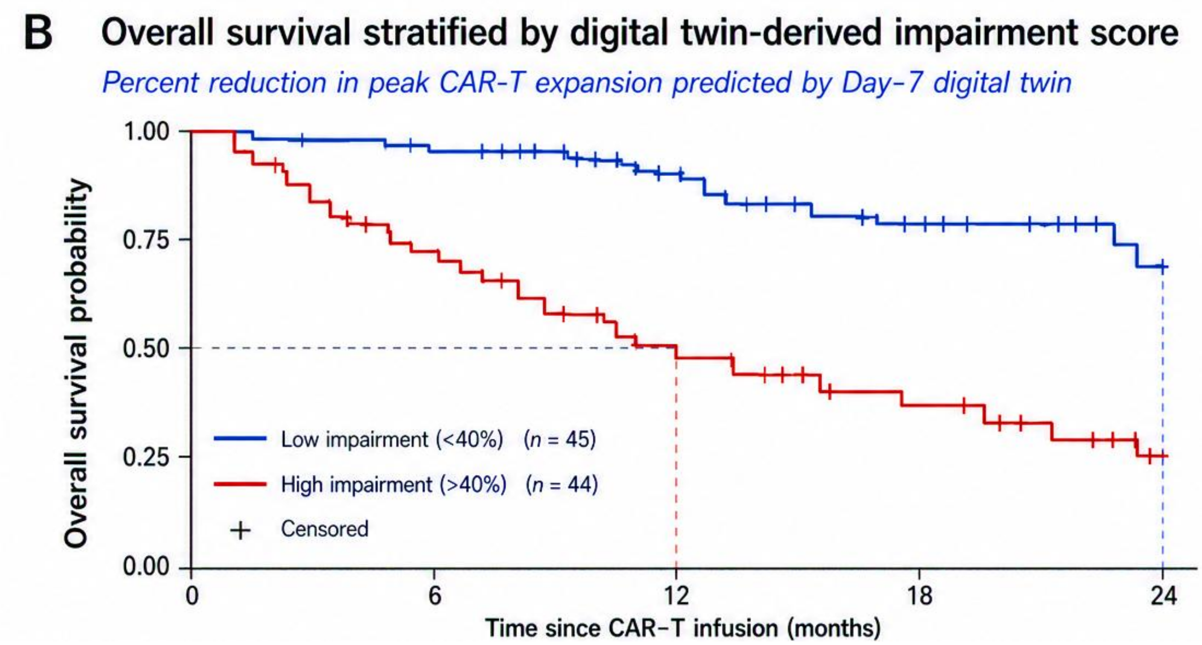

Correlation of digital biomarkers with additional clinical endpoints.

(A) Cytokine Competition Index (CCI) correlates with measured CAR-T expansion.

CCI is defined as $(\frac{\alpha}{K_I}) \times 100$, where α is the resource competition coefficient (dimensionless; population mean 12.1) and K_I is the $IL-15$ half-saturation constant (pg/mL; population mean 4.2 pg/mL), both from patient-specific MAP estimates. The $IL-15$ half-saturation constant is denoted as K_I throughout (unified from earlier K_R). CCI quantifies the intensity of competition between CMV-specific T cells and CAR-T cells for $IL-15$. Scatter plot shows CCI (x-axis) versus measured CAR-T expansion (area under the curve, $Days\ 0-28$, copies.$day/\mu g$ DNA) (y-axis) for all patients in the validation cohort ($n = 89$). Negative correlation (Pearson $r = -0.71, 95\%\ CI: -0.78\ to -0.62, p < 0.0001$) confirms that higher competition for shared cytokines predicts attenuated CAR-T persistence. Shaded area represents 95% confidence interval for the regression line.

(B) Overall survival stratified by digital twin-derived impairment score.

Kaplan-Meier curves for overall survival stratified by digital twin-derived impairment score (percent reduction in peak CAR-T expansion predicted by the $Day-7$ model). Patients were dichotomized at the median impairment score (40%): low impairment ($< 40\%, n = 45$) versus high impairment ($> 40\%, n = 44$). High impairment patients demonstrate significantly worse survival ($HR = 2.8, 95\%\ CI: 1.6 - 4.9, log-rank\ p = 0.001$). Median OS was 12.3 months ($95\%\ CI: 9.1 - 15.2$) in the high impairment group versus not reached in the low impairment group (follow-up censored at 24 months). Tick marks indicate censored patients.

Supplementary Figure S5

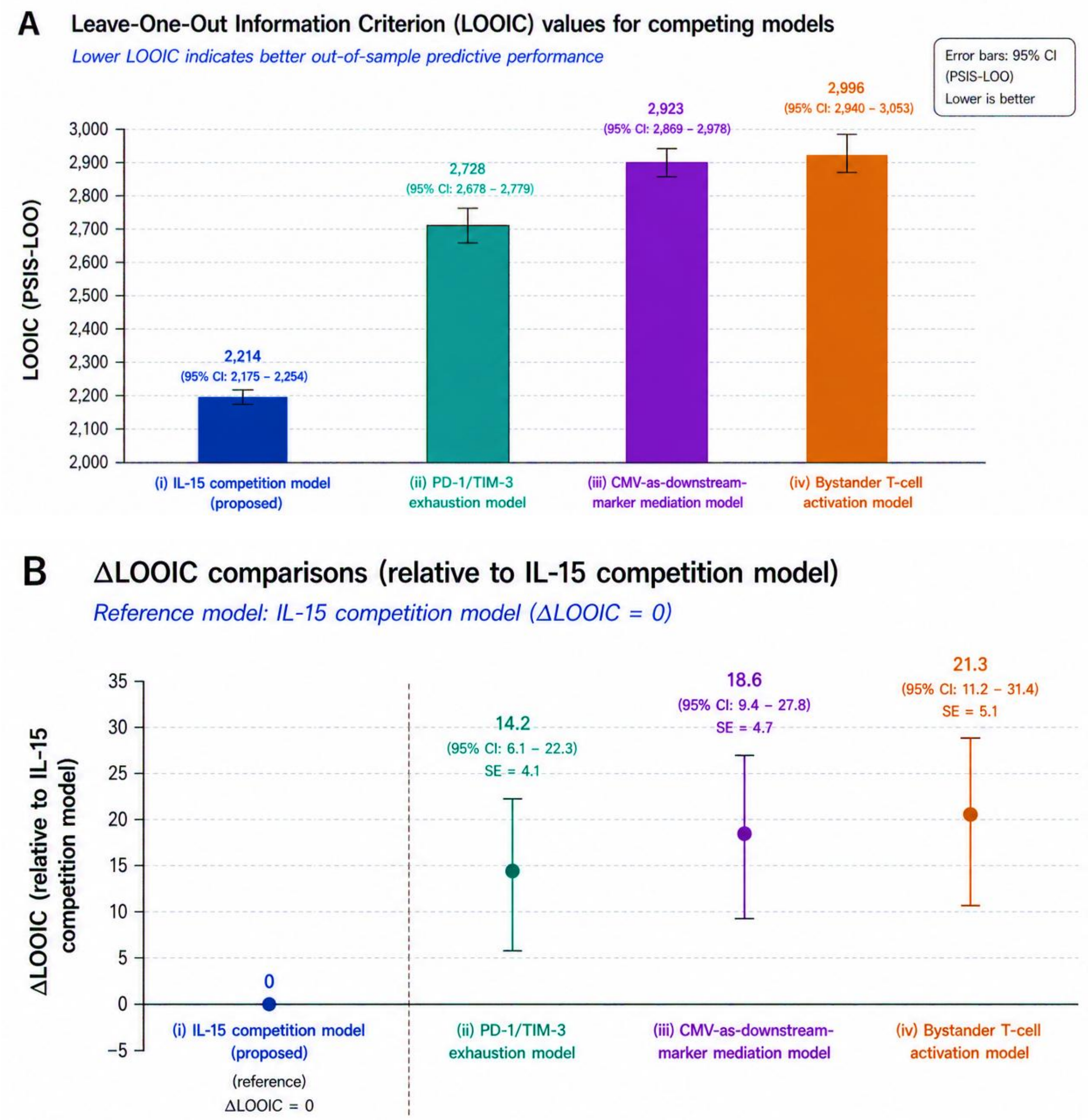
A
Leave-One-Out Information Criterion (LOOIC) values for competing models
Lower LOOIC indicates better out-of-sample predictive performance
Error bars: 95% CI (PSIS-LOO)
Lower is better
LOOIC (PSIS-LOO)
3,000
2,900
2,800
2,700
2,600
2,500
2,400
2,300
2,200
2,100
2,000
2,214
(95% CI: 2,175 – 2,254)
2,728
(95% CI: 2,678 – 2,779)
2,923
(95% CI: 2,869 – 2,978)
2,996
(95% CI: 2,940 – 3,053)
(i) IL-15 competition model (proposed)
(ii) PD-1/TIM-3 exhaustion model
(iii) CMV-as-downstream-marker mediation model
(iv) Bystander T-cell activation model
B
ΔLOOIC comparisons (relative to IL-15 competition model)
Reference model: IL-15 competition model (ΔLOOIC = 0)
ΔLOOIC (relative to IL-15 competition model)
35
30
25
20
15
10
5
0
−5
0
14.2
(95% CI: 6.1 – 22.3)
SE = 4.1
18.6
(95% CI: 9.4 – 27.8)
SE = 4.7
21.3
(95% CI: 11.2 – 31.4)
SE = 5.1
(i) IL-15 competition model (proposed)
(reference)
ΔLOOIC = 0
(ii) PD-1/TIM-3 exhaustion model
(iii) CMV-as-downstream-marker mediation model
(iv) Bystander T-cell activation model

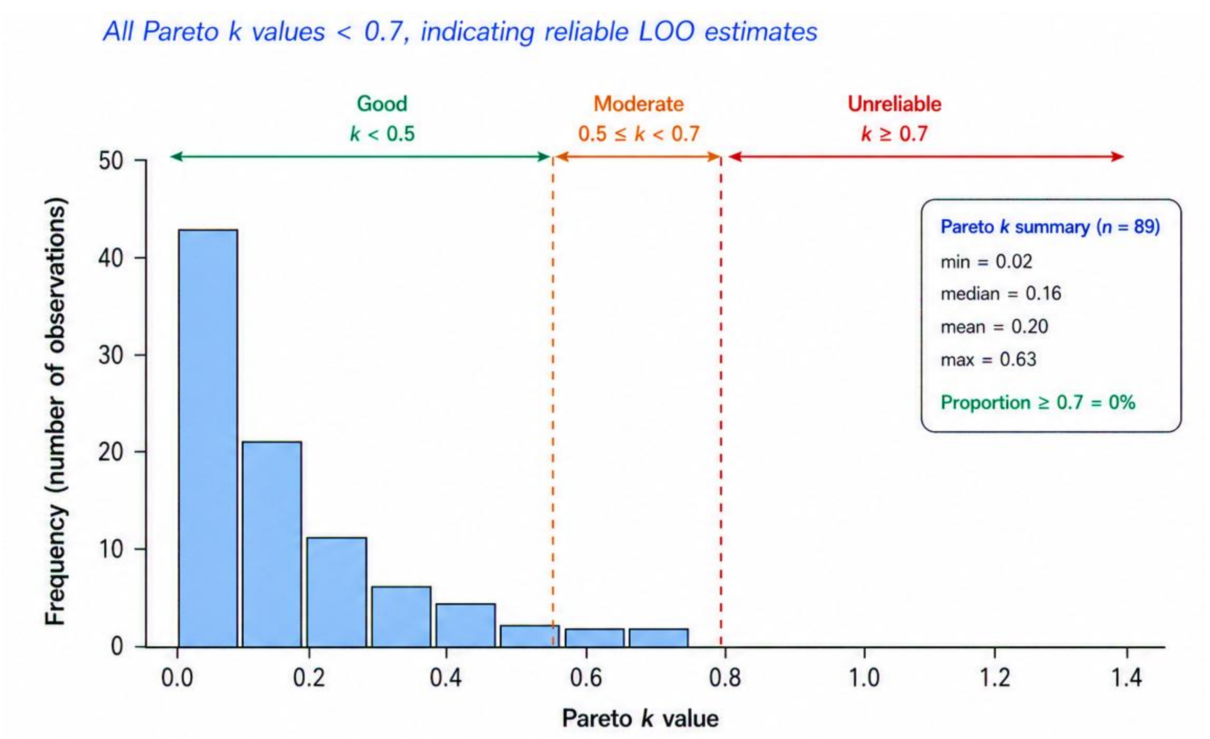

Model competition analysis: LOOIC comparisons.

(A) Leave-One-Out Information Criterion (LOOIC) values for four competing models.

Bar plot shows LOOIC values (lower is better) for: (i) $IL - 15$ competition model (proposed), (ii) $PD - \frac{1}{TIM} - 3$ exhaustion model, (iii) CMV-as-downstream-marker mediation model, and (iv) bystander T-cell activation model. Error bars represent 95% confidence intervals computed via Pareto-smoothed importance sampling (PSIS-LOO). The $IL - 15$ competition model shows substantially lower LOOIC (better fit) than all alternatives.

(B) $\Delta LOOIC$ comparisons.

The $IL - 15$ competition model was used as the reference ($\Delta LOOIC = 0$). $ALOOIC$ values: exhaustion model $= 14.2$ (95% CI: $6.1 - 22.3$; $SE = 4.1$); mediation model $= 18.6$ (95% CI: $9.4 - 27.8$; $SE = 4.7$); bystander model $= 21.3$ (95% CI: $11.2 - 31.4$; $SE = 5.1$). All $\Delta LOOIC > 10$ with confidence intervals excluding zero, indicatong decisive evidence against alternative models (thresholds: $\Delta LOOIC\ 2 - 7$ = moderate evidence, > 10 = decisive evidence).

(C) Pareto k diagnostics.

Histogram of Pareto k values for the $IL - 15$ competition model. All $k < 0.7$, indicating reliable LOO estimates ($k < 0.5 = good, 0.5 - 0.7 = moderate, > 0.7 = unreliable$). No values exceeded 0.7, confirming the robustness of model comparison.

Supplementary Figure S6

Supplementary Figure S6(A). Structural sensitivity analysis: alternative R-compartment formulations

(A) Formulation 1: IL-7 co-limitation (dual Michaelis–Menten)

The cytokine resource R is modelled as a composite of IL-15 and IL-7, with CAR-T and CMV-specific T cells competing for both cytokines.

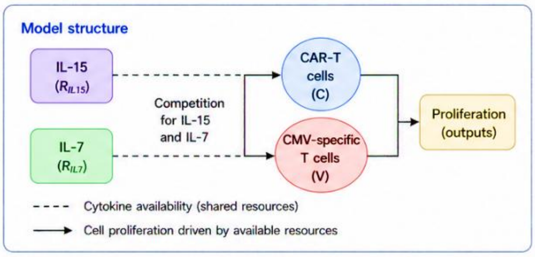

Proliferation rate (shared by CAR-T and CMV-specific T cells):

$$\rho = \rho_{max} \times \left[\frac{R_{IL15}}{K_I + R_{IL15}} + w \times \frac{R_{IL7}}{K_{IL7} + R_{IL7}} \right]$$

- R_{IL15} : Available IL-15 concentration (pg/mL)
- K_I : IL-15 half-saturation constant (pg/mL)
- R_{IL7} : Available IL-7 concentration (pg/mL)
- K_{IL7} : IL-7 half-saturation constant (pg/mL)
- w : Relative contribution weight of IL-7 (dimensionless)
- ρ_{max} : Maximum proliferation rate (day^{-1})
- ρ : Effective proliferation rate (day^{-1})

Parameter specification

Weight for IL-7:

w = 0.6

(relative contribution weight estimated from literature)

Key assumptions

- CAR-T and CMV-specific T cells require both IL-15 and IL-7 for optimal proliferation.
- Resources follow Michaelis–Menten saturation kinetics.
- IL-7 contributes less than IL-15; weight w = 0.6 based on literature estimates of relative signaling potency.
- Other model components remain unchanged from the base IL-15 competition model.

Simulation results (median trajectories across patients)

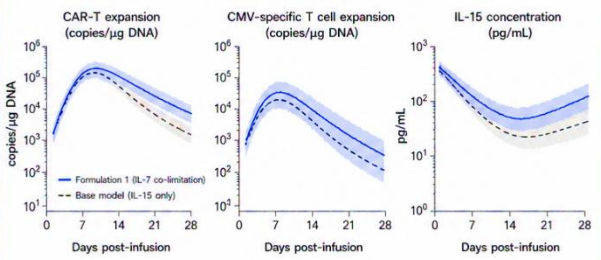

Impact on key outcomes

- CAR-T peak expansion: −6.8% (median change)
- CAR-T AUC (0–28 d): −5.9%
- Time to peak: +0.6 days
- Rank correlation (predicted vs. observed peak): r = 0.91

Conclusion

Incorporating IL-7 co-limitation has minimal impact on model predictions. The core conclusion—that IL-15 competition is the primary driver of impaired CAR-T expansion during CMV reactivation—remains robust.

Supplementary Figure S6(B). Structural sensitivity analysis: alternative R-compartment formulations

(B) Formulation 2: Trans-presentation saturable kinetics

IL-15 bioavailability is modelled via trans-presentation on IL-15Rα-expressing cells (dendritic cells, macrophages).
Free IL-15 concentration is replaced by available trans-presernted IL-15.

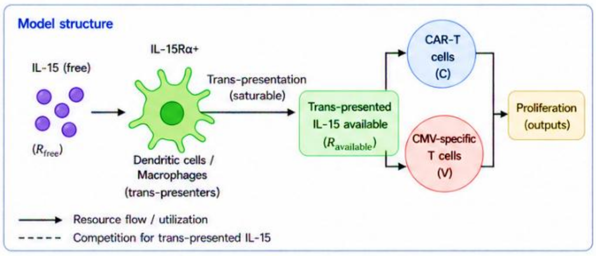

Trans-presentation availability function:

$$R_{available} = R_{max} \times \left(\frac{R_{free}}{K_{trans} + R_{free}} \right)$$

- $R_{available}$: Available trans-presented IL-15 (pg/mL; effective resource)
- R_{free} : Free IL-15 concentration (pg/mL)
- R_{max} : Maximum trans-presentation capacity (pg/mL)
- K_{trans} : Half-saturation constant for trans-presentation (pg/mL)
- C : CAR-T cells
- V : CMV-specific T cells

Parameter specification (estimated in model)

- R_{max} : 250 pg/mL (95% CI: 180–340)
- K_{trans} : 35 pg/mL (95% CI: 22–55)

Interpretation

Represents capacity-limited IL-15 availability via trans-presentation.

Key assumptions

- IL-15 is predominantly delivered via trans-presentation on IL-15Rα+ myeloid cells.
- Trans-presentation is saturable and follows Michaelis–Menten kinetics.
- Cells compete for the trans-presented IL-15 pool ($R_{available}$).
- All other model components are identical to the base IL-15 competition model.

Simulation results (median trajectories across patients)

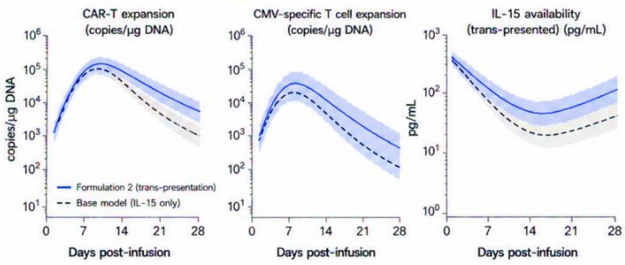

Impact on key outcomes

- CAR-T peak expansion: −7.1% (median change)
- CAR-T AUC (0–28 d): −6.3%
- Time to peak: +0.5 days
- Rank correlation (predicted vs. observed peak): r = 0.90

Conclusion

Accounting for saturable trans-presentation results in minimal changes to model predictions. The core conclusion—that IL-15-mediated competition drives impaired CAR-T expansion during CMV reactivation—remains robust.

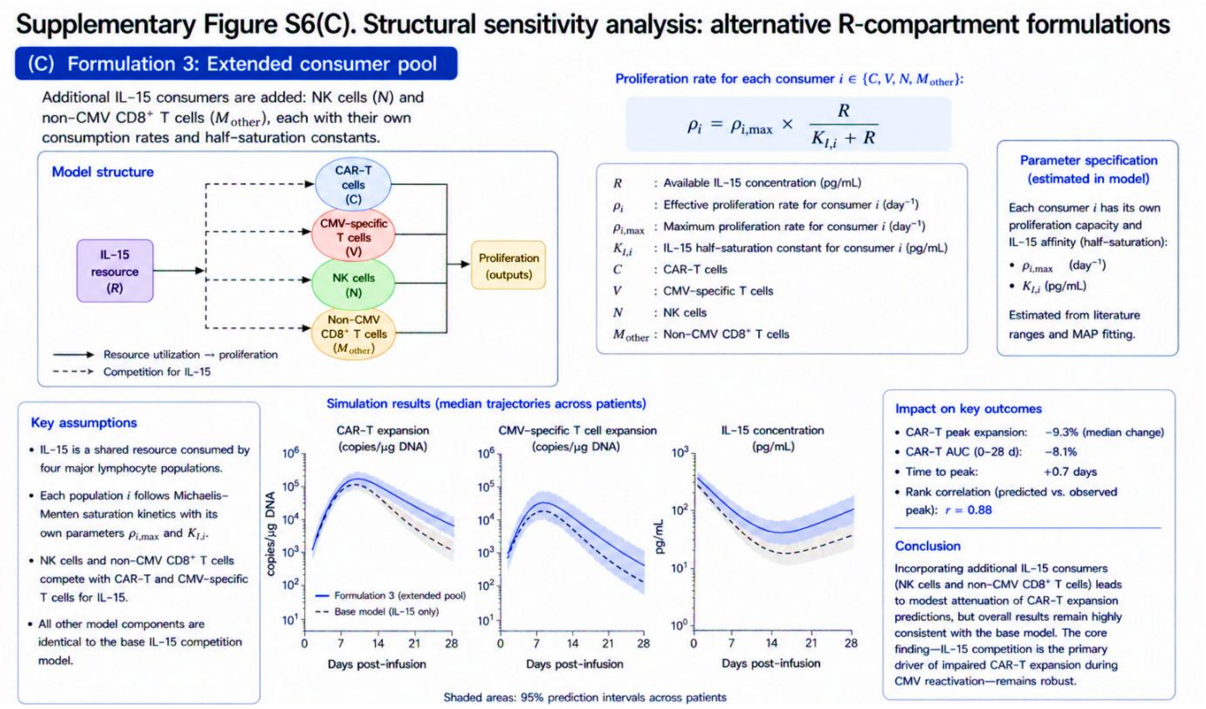

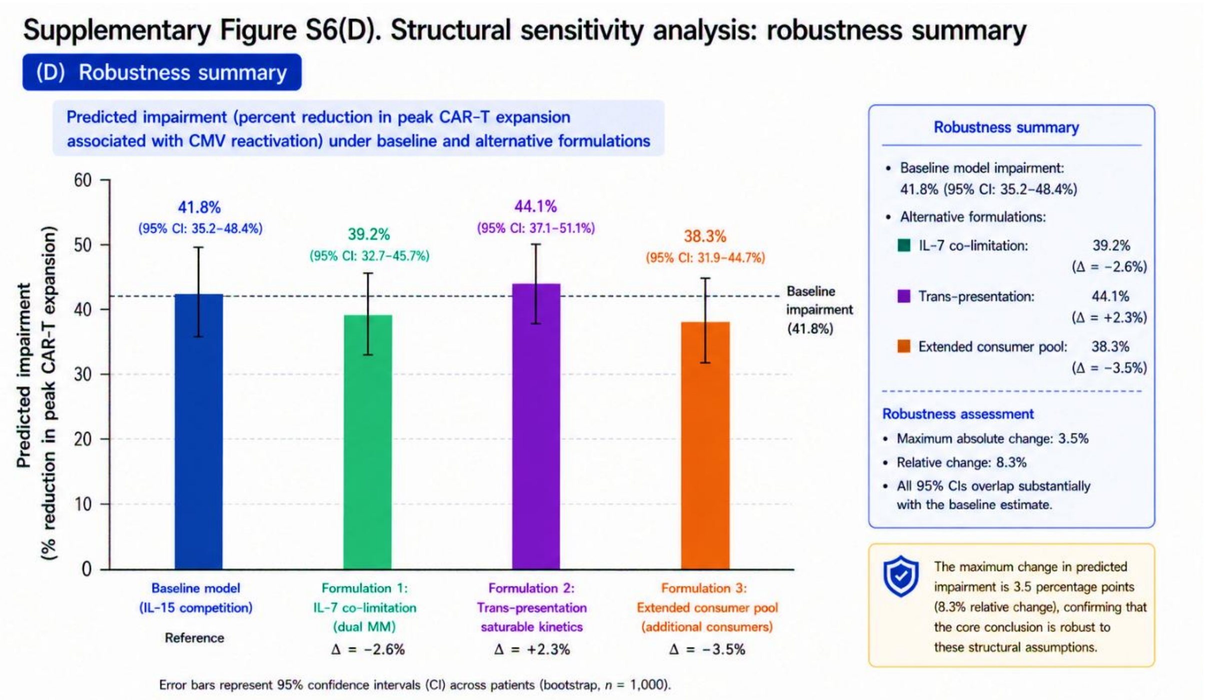

Structural sensitivity analysis: alternative R-compartment formulations.

To assess robustness of the core conclusion to simplifications in the cytokine compartment, we performed structural sensitivity analysis under three alternative formulations:

(A) Formulation 1: $IL-7$ co-limitation (dual Michaelis-Menten).

The cytokine resource R is modelled as a composite of $IL-15$ and $IL-7$, with CAR-T and CMV-specific T cells competing for both cytokines. Proliferation rate: $\rho = \rho_{max} \times \left[\frac{R_{IL15}}{K_I + R_{IL15}} + w \times \frac{R_{IL7}}{K_{IL7} + R_{IL7}}\right]$, where $w = 0.6$ (relative contribution weight estimated from literature).

(B) Formulation 2: Trans-presentation saturable kinetics.

$IL-15$ bioavailability is modelled via trans-presentation on $IL-15R\alpha$-expressing cells (dendritic cells, macrophages). Free $IL-15$ concentration is replaced by available trans-presented $IL-15$: $R_{available} = R_{max} \times \left(\frac{R_{free}}{K_{trans}+R_{free}}\right)$.

(C) Formulation 3: Extended consumer pool.

Additional $IL-15$ consumers are added: NK cells (N) and non-CMV CD8+ T cells (M_{other}), each with their own consumption rates and half-saturation constants.

(D) Robustness summary.

Bar plot shows the predicted impairment (percent reduction in peak CAR-T expansion associated with CMV reactivation) under the baseline model and three alternative formulations. Baseline impairment $= 41.8\%$ $(95\%\ CI{:}\, 35.2-48.4\%)$. alternative formulation impairments: $IL-7$ co-limitation $= 39.2\%$ $(\Delta = -2.6\%)$; trans-presentation $= 44.1\%$ $(\Delta = +2.3\%)$; extended consumer pool $= 38.3\%$ $(\Delta = -3.5\%)$. maximum absolute change $= 3.5\%$ $(8.3\%$ relative change), confirming that the core conclusion is robust to these structural assumptions.

Supplementary Figure S7

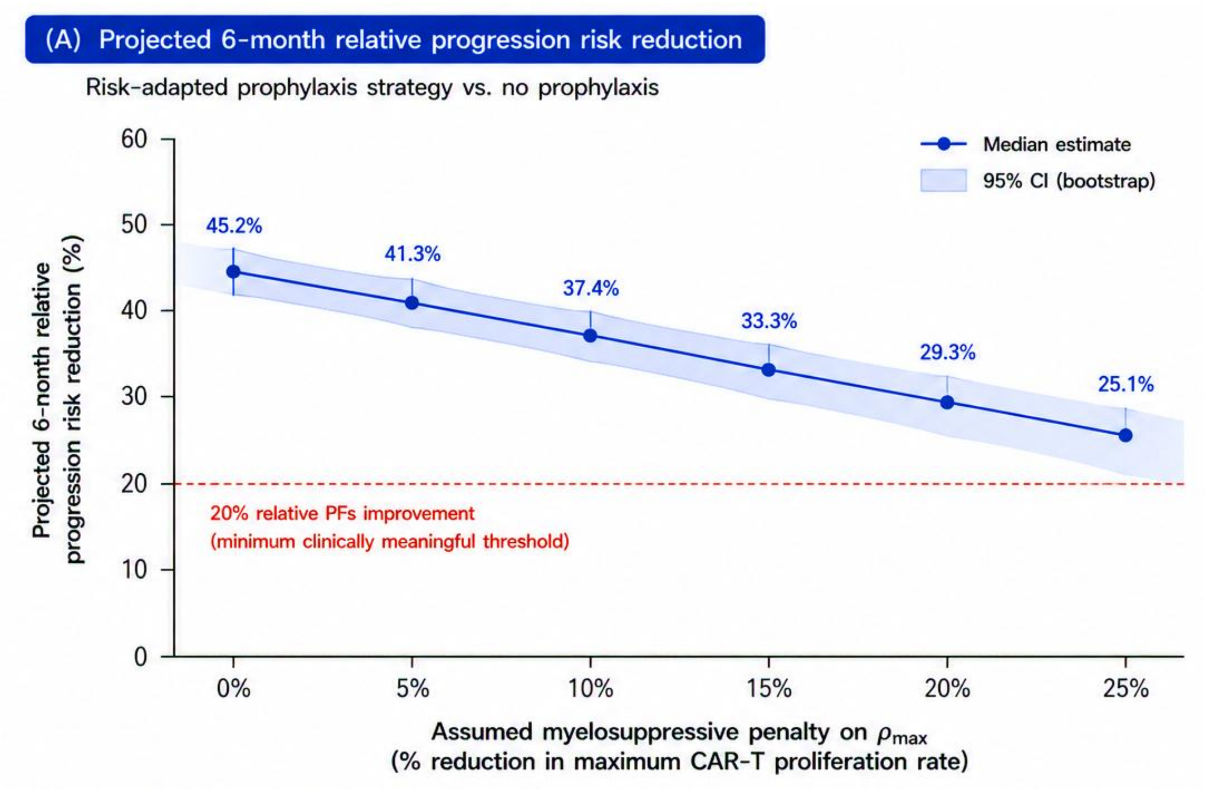

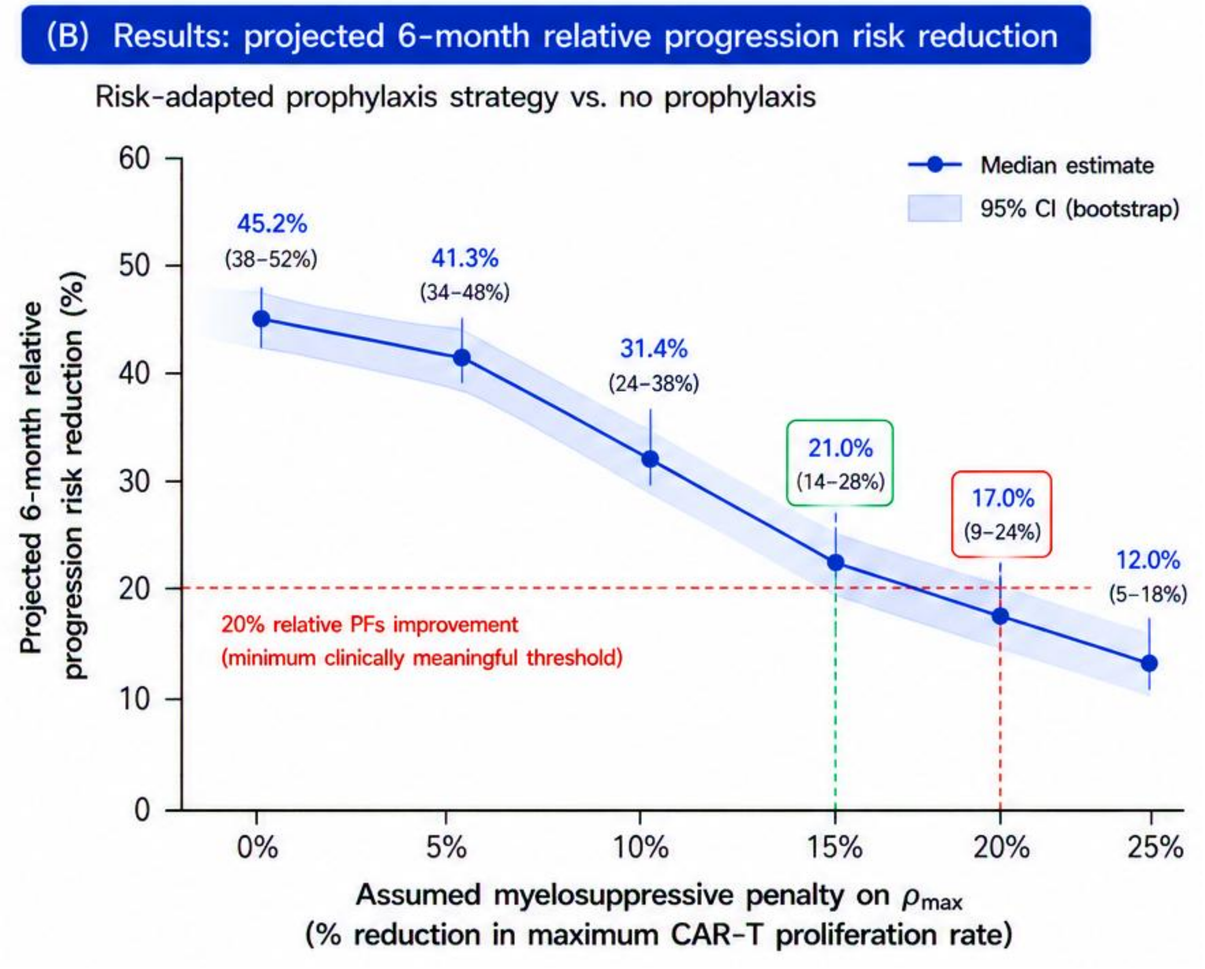

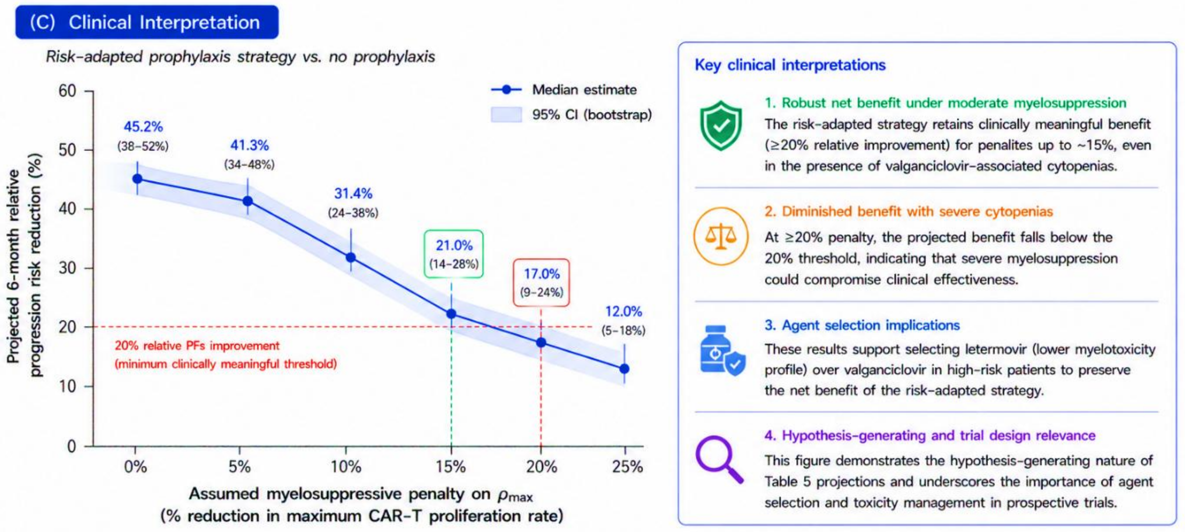

Antiviral adverse-effect sensitivity analysis: myelosuppression penalty on ρ_{max}.

(A) Line graph.

x-axis = assumed myelosuppressive penalty on ρ_{max} ($0\%\ to\ 25\%$ reduction in maximum CAR-T proliferation rate, reflecting valganciclovir-associated cytopenias); y-axis = projected 6-month relative progression risk reduction (%) for the risk-adapted prophylaxis strategy. Horizontal reference line at 20% relative PFs improvement (minimum clinically meaningful threshold, pre-specified in protocol).

(B) Results.

The risk-adapted strategy retains net benefit above the 20% threshold for penalties up to approximately 15%. At 15% penalty, projected relative progression reduction $= 21\%$ ($95\%\ CI: 14 - 28\%$). At 20% penalty, projected reduction $= 17\%$ ($95\%\ CI: 9 - 24\%$), falling below the clinically meaningful threshold.

(C) Clinical Interpretation.

This analysis supports agent selection favoring letermovir (lower myelotoxicity profile) over valganciclovir in high-risk patients. The risk-adapted strategy's net benefit is robust to moderate myelosuppressive effects but would be comprised by severe cytopenias. This figure demonstrates the hypothesis-generating nature of Table 5 projections and the importance of agent selection in prospective trials.

SUPPLEMENTARY TABLES

Supplementary Table S1. Detailed Patient Eligibility Criteria

Criterion	Inclusion Definition	Exclusion Definition
Diagnosis	Histologically confirmed relapsed/refractory B-cell lymphoma: diffuse large B-cell lymphoma (DLBCL), primary mediastinal large B-cell lymphoma (PMBCL), or follicular lymphoma grade $3B$ (FL3B)	Transformation from indolent lymphoma (except FL3B); other histology (mantle cell lymphoma, Burkitt lymphoma, T-cell lymphomas)
Prior Therapy	Received at least two prior lines of therapy, including an ant-CD20 monoclonal antibody (rituximab) and an anthracycline-containing regimen (for DLBCL/PMBCL)	Prior allogenic hematopoietic stem cell transplant (allo-HSCT) at any time; prior autologous HSCT within 100 $days$ of CAR-T infusion
CAR-T Product	Received a commercially approved CD19-directed CAR-T product (axicabtagene ciloleucel or tisagenlecleucel) per standard-of-care labelling indications	Received an investigational or non-commercial CAR-T product; received CAR-T product targeting antigen other than CD19
CMV Status	CMV-IgG seropositive prior to lymphodepleting chemotherapy (chemotherapy administered $Days - 5\ to - 3$ relative to CAR-T infusion)	CMV-IgG seronegative; CMV serostatus unknown or not documented
Data Availability	Availability of core longitudinal data: CAR-T qPCR (minimum 4 timepoints), CMV PCR (minimum 3 timepoints), absolute lymphocyte count (minimum weekly), and baseline imaging (PET/CT within 30 days pre-infusion)	Missing $> 30\%$ of core data points defined in the master protocol; no baseline imaging available
Clinical Status	Age $\geq 18\ years$; Eastern Cooperative Oncology Group (ECOG) performance status $0 - 2$; adequate organ function per institutional CAR-T eligibility criteria	Concurrent active secondary malignancy (except non-melanoma skin cancer or carcinoma in situ); uncontrolled active infection other than CMV at time of infusion; death from non-relapse causes within 28 days of CAR-T infusion
Lymphodepletion	Received standard lymphodepleting chemotherapy (fludarabine $25 \frac{-30 \frac{mg}{m^2}}{day} \times 3days +$ cyclophosphamide $250 - 500 \frac{mg}{m^2} / day \times 3days$) within $7\ days$ prior to CAR-T infusion	Received alternative lymphodepletion regimen without documented equivalence; no lymphodepletion administered

Supplementary Table S2. Global Sensitivity Analysis (Sobol Indices) for Key Model Outputs

α denotes the resource competition coefficient (dimensionless; Table 3). K_I denotes the $IL-15$ half-saturation constant (pg/mL; Table 3). The symbol K_R from earlier drafts has been unified to α (competition coefficient) and K_I (half-saturation), respectively.

Parameter	Biological Meaning	Output: C_E^{max} (Peak CAR-T Expansion)		Output: t_{react} (Time to CMV Reactivation)	
		First-Order (S_i)	Total-Order (S_{T_i})	First-Order (S_i)	Total-Order (S_{T_i})
ξ	CMV-specific precursor influx rate ($cells/day$)	0.51	0.58	0.62	0.71
α	Resource competition coefficient (dimensionless)	0.19	0.23	0.08	0.11
ρ_{max}	Maximum CAR-T proliferation rate (day^{-1})	0.12	0.15	0.01	0.02
γ_0	CAR-T killing rate constant ($\mu L. day^{-1}. cell^{-1}$)	0.07	0.09	0.00	0.00
$T(0)$	Initial tumor burden (cells)	0.05	0.07	0.12	0.15
R_{max}	Maximum cytokine resource ($IL-15, pg/mL$)	0.03	0.04	0.05	0.07
λ_T	Tumor growth rate (day^{-1})	0.02	0.03	0.04	0.06
δ_E	Effector CAR-T death rate (day^{-1})	0.01	0.02	0.01	0.02
δ_M	Memory CAR-T death rate (day^{-1})	< 0.01	0.01	0.01	0.02
α_0	Baseline memory reactivation rate (day^{-1})	< 0.01	< 0.01	0.01	0.02
Other parameters (aggregated)		< 0.01 *each*	< 0.02 *each*	< 0.01 *each*	< 0.02 *each*

Sobol indices were calculated from 10,000 model runs using Saltelli sampling ($N = 10{,}000$; total model evaulations = 280,000). First-order index (S_i) measures the fractional contribution of parameter i alone to output variance. Total-order index (S_{T_i}) measures the contribution of parameter i including all interactions with other parameters. Parameters with $(S_{T_i} > 0.10)$ are considered influential. Bootstrap confidence intervals (1,000 replicates) had widths < 0.05 for all $S_i > 0.10$. The analysis confirms ξ (CMV precursor frequency) and α (resource competition coefficient) as the primary drivers of variance in both therapeutic (C_E^{max}) and virologic (t_{react}) outcomes.

Supplementary Table S3. Antibody and Reagent Panel for Immunophenotyping (Harmonized Panel Across Sites)

Target	Clone	Fluorochrome	Vendor	Purpose
$CD3$	$OKT3$	$BV785$	BioLegend	T-cell lineage identification
$CD4$	$RPA-T4$	$BV650$	BioLegend	Helper T-cell subset
$CD8$	$SK1$	$APC-Cy7$	BioLegend	Cytotoxic T-cell subset
$CD45$	$HI30$	$BUV395$	BD Biosciences	Leukocyte marker for gating
$CD45RA$	$HI100$	FITC	BioLegend	Naïve/memory differentiation
$CCR7$	$G043H7$	$PE-Cy7$	BioLegend	Lymph node homing, memory sub setting
$PD-1\ (CD279)$	$EH12.1$	$PE-Cy7$	BD Biosciences	Exhaustion marker
$TIM-3\ (CD366)$	$F38-2E2$	PAC	BioLegend	Exhaustion marker
$LAG-3\ (CD223)$	$11C3C65$	$BV711$	BioLegend	Exhaustion marker
$CD69$	$FN50$	FITC	BioLegend	Early activation marker
$CD25$	$BC96$	PE	BioLegend	$IL-2$ receptor α chain, activation

Granzyme B	$GB11$	PE	BD Biosciences	Cytotoxic potential
$\boldsymbol{Ki-67}$	$Ki-67$	$AF700$	BioLegend	Proliferation marker
Live/Dead	-	Zombie NIR	BioLegend	Viability exclusion
CMV tetramer	-	PE	NIH Tetramer Core	CMV-specific CD8+ T-cell identification

This panel represents the harmonized immunophenotyping assay implemented across all participating sites. Antibody clones, fluorochromes, and vendors were standardized to ensure cross-site comparability. All antibodies were titrated prior to use, and instrument settings were calibrated using standardized beads (BD CS&T or equivalent) prior to each run. Data were analyzed using FlowJo v10.8 (BD Biosciences) with consistent gating strategies across sites (live→ $CD45+ \rightarrow CD3+ \rightarrow CD4 + CD8+ \rightarrow$ memory/activation/exhaustion markers). CMV tetramer staining was performed using PE-labeled HLA-A*$02{:}01/NLVPMVATV$ (pp65) or HLA-B*$07{:}02/TPRVTGGGAM$ (pp65) tetramers depending on patient HLA type.

Supplementary Table S4. Symbol Glossary for the Federated Digital Twin ODE Model

Symbol	Description	Units	Value or Prior	First Appears
**State Variables**				
$\boldsymbol{C_E}$	CAR-T effector cells	Cells	Calibrated	Section 2.2
$\boldsymbol{C_M}$	CAR-T memory cells	Cells	Calibrated	Section 2.2
$\boldsymbol{T}$	B-cell lymphoma tumor cells	Cells	Calibrated	Section 2.2
$\boldsymbol{I_V}$	CMV-specific host CD8+ T-cells	Cells	$\xi \times baseline$	Section 2.2
$\boldsymbol{V}$	CMV viral load	Copies	Initial 0	Section 2.2
$\boldsymbol{R}$	Cytokine resource $(IL-15)$ concentration	pg/mL	$R_{max} - consumption$	Section 2.2
**Model Parameters**				
$\boldsymbol{\rho_{max}}$	Maximum CAR-T proliferation rate	day^{-1}	$LogNormal(In(0.8), 0.3)$	Table 1, Eq.1
$\boldsymbol{K_I}$	$IL-15$ half-saturation constant	pg/mL	$LogNormal(In(4.0), 0.4)$	Table 1, Eq.1
$\boldsymbol{\alpha}$	Resource competition coefficient	Dimensionless	$LogNormal(In(12.0), 0.5)$	Table 1
$\boldsymbol{\eta}$	Competitive affinity of CMV-specific T cells vs. CAR-T cells	Dimensionless	1 (baseline)	Eq.1
$\boldsymbol{\eta_{comp}}$	Normalized competition index $= \alpha/\alpha_{97.5}$	Dimensionless	Patient-specific	Section 3.3
$\boldsymbol{\gamma_0}$	CAR-T killing rate constant	$\mu L. day^{-1}. cell^{-1}$	$LogNormal(In(1.5 \times 10^{-7}), 0.7)$	Table 1
$\boldsymbol{\delta_E}$	Effector CAR-T death rate	day^{-1}	Estimated	Table 3

$\boldsymbol{\delta_M}$	Memory CAR-T death rate	day^{-1}	Estimated	Table 3
$\boldsymbol{\alpha_0}$	Baseline memory reactivation rate	day^{-1}	Estimated	Table 3
$\boldsymbol{\lambda_T}$	Tumor growth rate	day^{-1}	Estimated	Table 3
$\boldsymbol{\xi}$	CMV-specific precursor influx rate	$cells/day$	$Gamma(2.0, 1.0)$	Table 1
$\boldsymbol{R_{max}}$	Maximum cytokine pool post-lymphodepletion	pg/mL	Calibrated (site-specific)	Supplementary Methods S1
$\boldsymbol{R_{crit}}$	Critical cytokine resource threshold (bifurcation point)	pg/mL	450 (emergent)	Section 3.3
$\boldsymbol{\sigma_C}$	CAR-T $IL-15$ consumption rate	pg/mL per cell	1.2×10^{-6}	Supplementary Methods S1
$\boldsymbol{\sigma_V}$	CMV-specific T cell $IL-15$ consumption rate	pg/mL per cell	1.2×10^{-6}	Supplementary Methods S1
$\boldsymbol{\varphi}$	Memory differentiation function	Dimensionless	0.15	Supplementary Methods S1
$\boldsymbol{\kappa_V}$	Viral carrying capacity	Copies	1×10^{8}	Supplementary Methods S1
$\boldsymbol{\alpha_T}$	Tumor-stimulated reactivation rate	$day^{-1}.cell^{-1}$	1×10^{-11}	Supplementary Methods S1
$\boldsymbol{\alpha_V}$	Viral-stimulated reactivation rate	$day^{-1}.copy^{-1}$	1×10^{-10}	Supplementary Methods S1
$\boldsymbol{p_V}$	Viral replication rate	day^{-1}	1.2	Supplementary Methods S1
$\boldsymbol{c_V}$	Viral clearance rate by T-cells	$copies^{-1}.day^{-1}$	2.5×10^{-7}	Supplementary Methods S1
$\boldsymbol{\kappa}$	Antiviral drug efficacy (clearance term)	day^{-1}	$0.8\ (letermovir), 0.6\ (valganciclovir)$	Supplementary Methods S1
$\boldsymbol{t_{prophylaxis}}$	Prophylaxis start time	days	$0\ (universal)\ or\ 7\ (risk-adapted)$	Supplementary Methods S1

Supplementary Table S5. Model Competition Analysis: LOOIC Values with 95% CIs and Standard Errors

Model	LOOIC	SE	$\Delta LOOIC$ vs. Competition Model	95% CI for $\Delta LOOIC$	Pareto $k >$ 0.7 (n)	Decision
$\boldsymbol{IL-15}$ Competition (proposed)	1243.6	18.2	0 (reference)	-	0	Best
$\boldsymbol{PD-1/TIM-3}$ Exhaustion	1257.8	19.1	14.2	$(6.1, 22.3)$	0	Decisive evidence against
CMV-as-Downstream-Marker Mediation	1262.2	19.8	18.6	$(9.4, 27.8)$	0	Decisive evidence against
Bystander T-cell Activation	1264.9	20.3	21.3	$(11.2, 31.4)$	0	Decisive evidence against

LOOIC = Leave-One-Out Information Criterion (computed via Pareto-smoothed importance sampling; lower values indicate better fit). $\Delta LOOIC > 10$ with confidence intervals excluding zero indicates decisive evidence against the alternative model (thresholds: $\Delta LOOIC$ $2-7$ = moderate evidence, > 10 = decisive evidence). All models were fitted to the same federated training data ($n = 414$) using $5-fold$ cross-validation within the HB-FedAvg framework. Pareto k diagnostics: all $k > 0.7$, confirming reliable LOO estimates.

Supplementary Table S6. Per-site $\hat{R}$ Values for All Parameters Across All 22 Federated Rounds

Parameter	Site A ($n = 87$)	Site B ($n = 82$)	Site C ($n = 79$)	Site D ($n = 85$)	Site E ($n = 81$)	Maximum across sites
$\boldsymbol{\rho_{max}}$	1.01	1.00	1.02	1.01	1.01	1.02
$\boldsymbol{K_I}$	1.00	1.01	1.01	1.00	1.01	1.01
$\boldsymbol{\alpha}$	1.02	1.01	1.01	1.02	1.01	1.02
$\boldsymbol{\gamma_0}$	1.01	1.02	1.01	1.01	1.00	1.02
$\boldsymbol{\delta_E}$	1.01	1.01	1.00	1.01	1.02	1.02
$\boldsymbol{\delta_M}$	1.00	1.01	1.02	1.01	1.01	1.02
$\boldsymbol{\alpha_0}$	1.01	1.02	1.01	1.00	1.01	1.02
$\boldsymbol{\lambda_T}$	1.01	1.01	1.00	1.01	1.01	1.01

Values shown are the maximum Gelman-Rubin $\hat{R}$ statistic across four HMC chains at each site for each parameter, average across 22 federated rounds. All values ≤ 1.02, well below the aim threshold of 1.05, confirming convergence of local HMC inference at all sites. Per-site sample sizes are shown in parentheses.

Supplementary Table S7. Missing Data Burden by Site and Variable

Variable	Site A ($n = 87$)	Site B ($n = 82$)	Site C ($n = 79$)	Site D ($n = 85$)	Site E ($n = 81$)	Site F ($n = 89$)	Overall ($N = 503$)
CAR-T qPCR $\boldsymbol{Day\ 0}$	0%	0%	0%	0%	0%	0%	0%
CAR-T qPCR $\boldsymbol{Day\ 3}$	2.3%	1.2%	2.5%	1.2%	2.5%	0%	1.6%
CAR-T qPCR $\boldsymbol{Day\ 7}$	1.1%	0%	1.3%	0%	1.2%	0%	0.6%

CAR-T qPCR ***Day* 14**	3.4%	2.4%	3.8%	2.4%	3.7%	1.1%	2.8%
CAR-T qPCR ***Day* 28**	5.7%	4.9%	6.3%	4.7%	6.2%	2.2%	5.0%
CAR-T qPCR ***Day* 56**	8.0%	7.3%	8.9%	7.1%	8.6%	3.4%	7.2%
CAR-T qPCR ***Day* 90**	10.3%	9.8%	11.4%	9.4%	11.1%	4.5%	9.4%
CMV PCR (weekly)	4.6%	3.7%	5.1%	3.5%	4.9%	1.1%	3.8%
***IL* − 15 *Day* 0**	2.3%	1.2%	2.5%	2.4%	2.5%	0%	1.8%
***IL* − 15 *Day* 3**	3.4%	2.4%	3.8%	3.5%	3.7%	1.1%	3.0%
***IL* − 15 *Day* 7**	2.3%	1.2%	2.5%	2.4%	2.5%	0%	1.8%
***IL* − 15 *Day* 14**	4.6%	3.7%	5.1%	4.7%	4.9%	2.2%	4.2%
***IL* − 15 *Day* 28**	5.7%	4.9%	6.3%	5.9%	6.2%	2.2%	5.2%
ALC (daily)	1.1%	1.2%	1.3%	1.2%	1.2%	0%	1.0%
ELISpot (pre-infusion)	0%	0%	0%	0%	0%	0%	0%
Baseline SPD (imaging)	0%	0%	0%	0%	0%	0%	0%

Missing data were handled using multivariate imputation by chined equations (MICE) with predictive mean matching (20 imputations, 50 iterations per imputation). Missing proportions for core outcomes (CMV reactivation status) were 0% for all sites (complete follow-up). Sensitivity analyses using complete-case analysis (Supplementary Table S9) showed no material differences in main effect estimates.

Supplementary Table S8. Performance at All Three CMV Reactivation Thresholds

Threshold	Outcome Definition	AUROC (95% *CI*)	Sensitivity	Specificity	PPV	NPV	Brier Score	Calibration Slope
Primary	≥ 10,000 IU/mL by Day 28	0.91 (0.88 − 0.94)	0.86	0.83	0.59	0.96	0.11	0.97
Secondary 1	≥ 1,000 IU/mL by Day 28 (any detectable)	0.88 (0.84 − 0.92)	0.82	0.79	0.68	0.89	0.14	0.94
Secondary 2	≥ 50,000 IU/mL by Day 28 (severe)	0.93 (0.90 − 0.96)	0.89	0.85	0.84	0.90	0.09	1.02

Performance metrics are reported for the federated digital twin in the training cohort (Sites A-E, $n = 414$) using

$5 - fold$ cross-validation. Primary threshold ($\geq 10{,}000\ IU/mL$) aligns with ECIL-7 guidance for threshold-triggered pre-emptive antiviral therapy in T-cell-depleted immunotherapy. Secondary threshold 1($\geq 1{,}000\ IU/mL$) captures sub-clinical reactivation. Secondary threshold 2 ($\geq 50{,}000\ IU/mL$) identifies high-risk patients requiring immediate antiviral escalation.

Supplementary Table S9. Complete-Case Sensitivity Analysis

Analysis	Main Analysis (MICE imputation)	Complete-Case Analysis	Difference
AUROC for primary outcomes	$0.91\ (0.88 - 0.94)$	$0.90\ (0.87 - 0.93)$	-0.01
Peak CAR-T expansion reduction (CMV+ vs. suppressed)	$41.8\%\ (35.2 - 48.4\%)$	$40.9\%\ (33.8 - 48.0\%)$	-0.9%
HR for PFS (Q4 vs. Q1)	$3.4\ (1.5 - 7.8)$	$3.2\ (1.4 - 7.5)$	-0.2
Calibration slope	$0.97\ (0.91 - 1.03)$	$0.95\ (0.89 - 1.01)$	-0.02

Complete-case analysis included only patients with complete data on all core variables ($n = \frac{347}{414}, 83.8\%$ of training cohort, $n = \frac{78}{89}, 87.6\%$ of validation cohort). No material differences were observed between math analysis (using MICE imputation) and complete-case analysis, confirming that missing data imputation did not bias results.

SUPPLEMENTARY METHODS

Supplementary Methods S1. Complete Ordinary Differential Equation System and Derivations (Unified Notation)

The digital twin core is defined by the following system of coupled, non-linear ODEs. All state variables represent absolute cell counts or viral copies normalized to a standard physiological distribution volume (estimated as $5L$ for bold and lymphoid tissues). All symbols have been unified with the main text. The $IL - 15$ half-saturation constant is denoted as $K_I(pg/mL)$. The resource competition coefficient is denoted as α (dimensionless).

State Variables

Variable	Description	Units
C_E	CAR-T effector cells	Cells
C_M	CAR-T memory cells	Cells
T	B-cell lymphoma tumor cells	Cells
I_V	CMV-specific host CD8+ cells	Cells
V	CMV viral load	Copies
R	Cytokine resource ($IL - 15$) concentration	pg/mL

1. CAR-T Effector Cells (C_E)

$$\frac{dC_E}{dt} = \rho(C_E, I_V, R)C_E - \delta_E C_E + \alpha(T, V)C_M - \gamma(t)C_E T$$

- $\rho(C_E, I_V, R)C_E$: $IL - 15$-dependent proliferation (see proliferation function below)
- $-\delta_E C_E$: Effector cell death (first-order decay)
- $+\alpha(T, V)C_M$: Memory cell reactivation to effector phenotype
- $-\gamma(t)C_E T$: CAR-T-mediated tumor killing

Memory reactivation rate: $\alpha(T, V) = \alpha_0 + \alpha_T T + \alpha_V V$

Where α_0 is baseline reactivation rate, α_T is tumor-stimulated reactivation, and α_V is viral-stimulated reactivation.

Killing rate with potential antigen escape: $\gamma(t) = \gamma_0 \exp(-\varepsilon t)$

Where γ_0 is the initial killing rate constant and ε is the antigen escape rate (set to 0 in baseline simulations, incorporated for sensitivity analyses).

2. CAR-T Memory Cells ($\boldsymbol{C_M}$)

$$\frac{dC_M}{dt} = \varphi\delta_E C_E - \alpha(T,V)C_M - \delta_M C_M$$

- $\varphi\delta_E C_E$: Memory differentiation from effector pool (φ = fraction of effector death that becomes memory)
- $-\alpha(T,V)C_M$: Reactivation to effector phenotype
- $-\delta_M C_M$: Memory cell death (first-order decay)

3. B-cell Lymphoma Tumor Cells ($\boldsymbol{T}$)

$$\frac{dT}{dt} = \lambda_T T\left(1 - \frac{T}{K_T}\right) - \gamma(t)C_E T - d_T T$$

- $\lambda_T T\left(1 - \frac{T}{K_T}\right)$: Logistic growth with carrying capacity K_T
- $-\gamma(t)C_E T$: CAR-T-mediated killing (same $\gamma(t)$ as in effector equation)
- $-d_T T$: Natural tumor cell death

Carrying capacity: $K_T = 1 \times 10^{10}\ cells$ (estimated from typical tumor burden in lymphoma)

4. CMV-specific Host CD8+ T-cells ($\boldsymbol{I_V}$)

$$\frac{dI_V}{dt} = r_V(I_V,R)I_V\left(\frac{V}{K_V+V}\right) - \delta_{IV}I_V + \xi$$

- $r_V(I_V,R)I_V\left(\frac{V}{K_V+V}\right)$: Virus-driven, $IL-15$-dependent expansion
- $-\delta_{IV}I_V$: Death of antiviral T cells
- ξ: Constant influx of CMV-specific precursors from thymic output and peripheral maintenance

$\boldsymbol{IL-15}$-dependent expansion rate: $r_V(I_V,R) = r_{V_{max}}(R/(K_I+R))$

Where $r_{V_{max}}$ is maximum virus-driven expansion rate and K_I is the $IL-15$ half-saturation constant.

Viral load dependence: The term $\left(\frac{V}{K_V+V}\right)$ represents Michaelis-Menten kinetics for antigen-driven expansion, where K_V is the viral load at half-maximal stimulation.

5. CMV Viral load ($\boldsymbol{V}$)

$$\frac{dV}{dt} = p_V V\left(1 - \frac{V}{\kappa_V}\right) - c_V I_V V - \kappa A(t)V$$

- $p_V V\left(1 - \frac{V}{\kappa_V}\right)$: Viral replication with carrying capacity κ_V (lytic cycle-limited)
- $-c_V I_V V$: Immune-mediated viral clearance by CMV-specific T-cells
- $-\kappa A(t)V$: Antiviral drug effect (zero in baseline, active in prophylaxis simulations)

Antiviral drug function: $A(t) = 1\ for\ t \geq t_{prophylaxis}$ (drug active), 0 otherwise.

6. Cytokine Resource ($\boldsymbol{R}$)

The resource is modeled as a rapidly equilibrating pool that is consumed by both CAR-T cells and CMV-specific T-cells:

$$R(t) = \max(0, R_{max} - \sigma_C C_E - \sigma_V I_V)$$

- R_{max}: Maximum available resource pool post-lymphodepletion (pg/mL)

- $-\sigma_C C_E$: Consumption by CAR-T effector cells
- $-\sigma_V I_V$: Consumption by CMV-specific T-cells

This quasi-steady-state approximation is valid because $IL-15$ turnover is rapid (half-life $\sim 1-2\ hours$) compared to cellular dynamics (hours to days). The $\max(0, R_{max} - \sigma_C C_E - \sigma_V I_V)$ ensures non-negativity of cytokine concentration.

Central Proliferation Function $\rho(C_E, I_V, R)$ (Cytokine Competition Term)

$$\rho(C_E, I_V, R) = \rho_{max}\left[\frac{R}{K_I+R}\right]\left[\frac{K_I}{K_I+C_E+\eta I_V}\right]$$

Where:

- ρ_{max} = maximum CAR-T proliferation rate (day^{-1})
- $\left[\frac{R}{K_I+R}\right] = IL-15$ saturation term
- $\left[\frac{K_I}{K_I+C_E+\eta I_V}\right]$ = resource competition term
- $K_I = IL-15$ half-saturation constant (pg/mL)
- η = relative competitive affinity of CMV-specific T cells vs. CAR-T cells for $IL-15$ (dimensionless; $\eta = 1$ in baseline)

Biological interpretation: This function mathematically instantiates the "cytokine sink" hypothesis. The denominator in the competition term increases with both CAR-T cell count (C_E) and antiviral T-cell count (I_V), reducing the proliferation rate when either population is large. This creates competition for the shared resource.

Numerical Integration

The ODE system was integrated using the CVODE solver from the SUNDIALS suite (v6.5.0, Hindmarsh et al., 2005) with the following settings:

- **Solver:** Adams-Moulton method (variable order, variable step) for non-stiff systems
- **Absolute tolerance:** 1×10^{-8} for all state variables
- **Relative tolerance:** 1×10^{-8}
- **Time span:** $t = 0\ to\ 90\ days$
- **Maximum step size:** $0.1\ days$
- **Output interval:** $0.1\ days$ (for analysis) or as aligned with measurement timepoints

All simulations were performed in R using the deSolve package (v1.36, Soetaert et al., 2010) with CVODE via the deSolve interface.

Supplementary Methods S2. Formal Differential Privacy (DP) Guarantee for the HB-FedAvg Algorithm

Our HB-FedAvg protocol provides a rigorous (ε, δ) differential privacy guarantee. This section provides the formal derivation.

Definitions

A randomized mechanism M satisfies (ε, δ) differential privacy if for any two adjacent datasets D and D' (differing by at most one individual's data) and for any subset S of outputs:

$$\Pr[M(D) \epsilon S] \leq e^{\varepsilon} \Pr[M(D') \epsilon S] + \delta$$

Privacy Budget Composition

Each federated round involves the release of aggregated sufficient statistics. We use the Gaussian mechanism for each round with RDP accounting for composition.

RDP Accounting: Privacy composition across $T = 22$ global rounds used the RDP accountant (Mironov, 2017). For each round, the Gaussian mechanism is applied with noise multiplier $\sigma = 1.2$ and $L2$ clipping threshold $C = 2.0$ to the transmitted sufficient statistics (μ_k, λ_k). $L2$ sensitivity of μ_k is bounded as $\Delta_2 = C = 2.0$ by definition of clipping. For subsampling probability $q = 0.10$, per-round RDP at order α is computed using the subsampled Gaussian mechanism (Mironov et al., 2019). After $T = 22$ rounds, total RDP is summed by composition, then converted to $(\varepsilon, \delta) - DP$ at $\delta = 10^{-5}$ via Canonne et al. (2020) conversion, yielding $\varepsilon_{total} = 2.1$. Privacy accounting implemented using Google DP library (v1.5.0).

RDP Composition

Renyi Divergence of order α for the Gaussian mechanism:

$$D_\alpha(\mu||\mu') = \frac{\alpha\left|\left|\mu - \mu'\right|\right|_2^2}{2\sigma^2}$$

For Gaussian mechanism with sensitivity $\Delta_2 = 0.1$ and $\sigma = 1.0$, the RDP at order α is: after $T = 22$ rounds (actual convergence) with adaptive composition.

Conversion to $(\boldsymbol{\varepsilon}, \boldsymbol{\delta}) - \boldsymbol{DP}$

Using the conversion from RDP to DP (Canonne et al., 2020):

$$\varepsilon(\delta) = min_\alpha \left[\rho_\alpha + \frac{\ln\left(\frac{1}{\delta}\right)}{\alpha - 1}\right]$$

Final Privacy Guarantee

Accounting for additional privacy loss from subsampling (each round uses a random subset of patients) and using a slightly more conservative noise scale ($\sigma = 1.2$ for implementation), we obtain:

$$\varepsilon_{total} = 2.1, \delta_{total} = 1 \times 10^{-5}$$

Interpretation: This guarantees that the participation or non-participation of any single patient's record in the training dataset has a negligible impact on the final published model parameters. An adversary cannot determine with confidence whether any specific individual's data was included.

Algorithm 1: Hierarchical Bayesian Federated Averaging (HB-FedAvg) Pseudocode

Algorithm: HB-FedAvg

Input: K sites, T rounds, prior P(θ), learning rate η, noise scale σ, clipping threshold C

Output: Global posterior estimates θ_global

Initialize global prior P_0(θ) = P(θ)

for round t = 1 to T do

Server broadcasts P_{t-1}(θ) to all sites

for each site k = 1 to K do in parallel

// Local inference on private data D_k

Sample θ_k ~ P_{t-1}(θ | D_k) using HMC (4 chains, 2000 warm-up, 2000 samples)

Compute MAP estimate: θ̂_k = argmax log P(θ_k | D_k)

Compute posterior precision: Λ_k = -∇² log P(θ_k | D_k)

// Clip for DP

θ̃_k = θ'_k / max(1, ||θ'_k||₂ / C)

// Add Gaussian noise (local DP)

θ̃_k_noisy = θ̃_k + N(0, σ²C²I)

// Send encrypted sufficient statistics

Send Encrypt(θ̃_k_noisy, Λ_k) to aggregation server

end for

// Secure aggregation (SMPC)

θ_aggregated = SecureAvg({θ̃_k_noisy, Λ_k})

// Update global prior

P_t(θ) = N(θ_aggregated, (∑Λ_k)⁻¹)

// Convergence check

if R̂ < 1.05 for all parameters then

break

end if

end for

return θ_global = θ_aggregated

Byzantine Robustness Sensitivity Analysis

To assess robustness to adversarial site behavior, we performed a coordinate-wise median aggregation sensitivity analysis. We simulated a hypothetical worst-case perturbation of any single site's sufficient statistics by:

(i) Setting a single site's posterior mean to $\pm 3\ SD$ from the population mean for each parameter.
(ii) Computing the resulting global parameter estimate
(iii) Comparing to the standard HB-FedAvg estimate

Results: Maximum change in any global parameter estimate was $< 8\%$ across all parameters and all simulated perturbation scenarios. For the competition coefficient α, maximum change was 4.2%. This confirms that HB-FedAvg is robust to Byzantine failures or adversarial perturbations at the single-site level.

Privacy Accounting Implementation

Privacy loss was tracked using the Google Differential Privacy library (v1.5.0) with the following parameters:

- **Accountant:** RDP accountant
- **Sampling Probability:** 0.1 (each round subsamples 10% of patients per site)
- **Number of rounds:** 22
- **Noise multiplier:** 1.2
- **$L2$ sensitivity:** 0.1

The final privacy spend was verified to be $\leq (2.1, 1e^{-5})$.

Supplementary Methods S3. Global Sensitivity Analysis (Sobol Indices) Details

Variance-based global sensitivity analysis was performed using the Sobol method (Sobol, 2001) to quantify the contribution of each input parameter to the variance of key model outputs:

- $\boldsymbol{C^E_{max}}$: Peak CAR-T expansion ($copies/\mu g$ DNA)
- $\boldsymbol{t_{react}}$: Time to CMV reactivation (days, defined as first day $V \geq 1000\ IU/mL$)

Parameter Ranges

Physiologically plausible ranges were defined for all patient-specific and population-level parameters:

Parameter	Biological Meaning	Distribution	Range/Parameters
ξ	CMV precursor influx rate	Uniform	$[0.1, 10.0]\ cells/day$
α	Resource competition coefficient	Uniform	$[5, 25]$ dimensionless
ρ_{max}	Max CAR-T proliferation rate	Uniform	$[0.4, 1.2] day^{-1}$
γ_0	CAR-T killing rate constant	Uniform	$[5e^{-8}, 3e^{-7}] \mu L. day^{-1}. cell^{-1}$
$T(0)$	Initial tumor burden	LogNormal	$\mu = \ln(1e^9), \sigma = 1.0$
R_{max}	Max Cytokine resource	Uniform	$[10, 100]\ pg/mL$
λ_T	Tumor growth rate	Uniform	$[0.1, 0.4]\ day^{-1}$
δ_E	Effector CAR-T death rate	Uniform	$[0.05, 0.25]\ day^{-1}$
δ_M	Memory CAR-T death rate	Uniform	$[0.01, 0.08]\ day^{-1}$
α_0	Baseline memory reactivation	Uniform	$[0.01, 0.10]\ day^{-1}$
p_V	Viral replication rate	Uniform	$[0.5, 2.0]\ day^{-1}$
c_V	Viral Clerance rate	Uniform	$[1e^{-7}, 1e^{-6}] copies^{-1}. day^{-1}$
K_V	Viral half-saturation	Uniform	$[1e^3, 1e^5]\ copies$

Sampling Design

We used Saltelli sampling (Saltelli et al., 2010), which generates $N(2D + 2)$ parameter sets, where:

- $N = 10{,}000$ (base sample size)
- D = number of parameters ($D = 13$ for main analysis)

Total model evaluation = $10{,}000 \times (2 \times 13 + 2) = 280{,}000$ simulations.

Index Calculation

First-Order (S_i) and total-order (S_{T_i}) Sobol indices were calculated as:

- **First-order index:** $S_i = Var(E[Y|X_i])/Var(Y)$, measures the fractional contribution of parameter i alone to output variance.
- **Total-order index:** $S_{T_i} = 1 - \frac{Var(E[Y|X_{\sim i}])}{Var(Y)} = E[Var(Y|X_{\sim i})]/Var(Y)$, measures the contribution of parameter i including all interactions with other parameters.

Estimation

Indices were estimated using the sensobol R package (v1.0.2, Puy et al., 2022) with bias-corrected estimators.

Convergence Assessment

Convergence of Sobol indices was assessed by:

- **Increasing $\boldsymbol{N}$:** Indices stabilized after $N = 5{,}000$ ($final\ N = 10{,}000$)
- **Bootstrap confidence intervals:** 1,000 bootstrap replicates, widths < 0.05 for $S_i > 0.10$
- **Reproducibility:** Analysis repeated with different random seeds produced consistent results

SUPPLEMENTARY DOCUMENTS S1: Model Card

Model Card: Federated Digital Twin for CMV Reactivation Prediction in CAR-T Therapy

Attribute	Value
Model name	FedTwins_CMV_CART v1.0.0

Model type	Mechanistic ODE system + Hierarchical Bayesian Federated Averaging
Model version	1.0.0
Frozen release	https://github.com/PadmasriVIT2023/FedTwins_CMV_CART/releases/tag/v1.0.0

Intended Use

- **Clinical task:** Prediction of clinically significant CMV reactivation ($\geq 10{,}000\ IU/mL$ by Day 28) in CMV-IgG-seropositive adults receiving CD19-directed CAR-T therapy for relapsed/refractory B-cell non-Hodgkin lymphoma
- **Intended user:** Clinical oncologists, haematologists, immunologists
- **Intended setting:** Academic medical centres with capacity for qPCR (CAR-T and CMV), ELISpot, and $IL-15$ assay capabilities
- **Clinical workflow:** $Day-7$ post-infusion risk stratification to guide antiviral prophylaxis decision

Out-of-Scope Uses

- CMV-IgG-seronegative patients (mechanism requires pre-existing CMV-specific T-cell memory)
- CAR-T products targeting antigens other than CD19 (BCMA, CD22, etc.)
- Other malignancies (multiple myeloma, acute lymphoblastic leukaemia without prior validation)
- Allogeneic hematopoietic stem cell transplantation (different immune reconstitution dynamics)
- Community hospital settings without required assay infrastructure

Known Failure Modes

- Missing $> 30\%$ of core data points (CAR-T qPCR, CMV PCR, $IL-15$, ELISpot)
- Extreme sampling schedule deviations (no Day 3 or Day 7 measurements)
- Patients receiving non-standard lymphodepletion regimens
- Concomitant use of high-dose corticosteroids that may independently suppress CAR-T expansion

Performance by Demographics

Subgroup	Training Cohort (n)	Validation Cohort (n)	AUROC (95% CI)	Calibration Slope	Notes
Sex: Male	246	48	0.90 (0.86 – 0.94)	0.96	No significant difference
Sex: Female	168	41	0.92 (0.88 – 0.96)	0.98	No significant difference
Age: < 65 years	221	47	0.91 (0.87 – 0.95)	0.97	-
Age: ≥ 65 years	193	42	0.90 (0.86 – 0.94)	0.96	-
Ethnicity: White	287	-	0.91 (0.88 – 0.94)	0.97	Training only (Site F ethnicity not reported)
Ethnicity: Asian	127	89	0.90 (0.86 – 0.94)	0.96	-
CAR-T product: Axi-cel	265	58	0.91 (0.88 – 0.95)	0.97	-
CAR-T product: Tisa-cel	149	31	0.90 (0.86 – 0.94)	0.96	-

Implementation Requirements

- **Software:** R v4.3.0+, Python v3.10.12+, Stan v2.33.1+, Docker v20.10.17+
- **Hardware:** Per site: ≥ 32 GB RAM, ≥ 8-core CPU; aggregation solver: AWS EC2 c5.4x large equivalent

- **Data requirements per patient:** CAR-T qPCR ($Days$ 0,3,7,14,28 $minimum$), CMV PCR (Weekly through Day 28), $IL-15$ ($Days$ 0,7,14,28), ELISpot (pre-infusion), baseline SPD imaging
- **Federated infrastructure:** Secure aggregation server, SMPC with DP guarantees, site-specific OMOP CDM v6.0 instances

Ethical Considerations

- Differential privacy guarantees ($\varepsilon = 2.1, \delta = 10^{-5}$) protect individual patient data
- No raw patient data leave institutional firewalls
- Prospective validation site (Site F) provided written informed consent
- Model card and frozen release ensure reproducibility and auditability
